# Impact of Various Essential Oils or Their Pure Components on the Selected Properties and Microbicidal Potential of Chitosan-Based Coatings

**DOI:** 10.1021/acs.langmuir.6c00099

**Published:** 2026-03-16

**Authors:** Mikołaj Mielczarek, Jakub Marchewka, Alicja Łukaszczyk, Katarzyna Biegun-Drożdż, Kamil Drożdż, Tomasz Gosiewski, Maciej Sitarz, Monika Brzychczy-Włoch, Tomasz Moskalewicz

**Affiliations:** † Faculty of Metals Engineering and Industrial Computer Science, 49811 AGH University of Krakow, Czarnowiejska 66, 30-054 Kraków, Poland; ‡ Faculty of Materials Science and Ceramics, AGH University of Krakow, Mickiewicza Av. 30, 30-059 Kraków, Poland; § Faculty of Foundry Engineering, AGH University of Krakow, Reymonta 23, 30-059 Kraków, Poland; ∥ Faculty of Medicine, Chair of Microbiology, Department of Molecular Medical Microbiology, 49573 Jagiellonian University Medical College, Czysta 18, 31-121 Kraków, Poland

## Abstract

Novel chitosan coatings incorporating antibacterial additives, including essential oils (cinnamon bark, cinnamon leaf, and thyme) or their constituents (cinnamaldehyde, eugenol, thymol, and carvacrol), were electrophoretically deposited on titanium. The electrokinetic characteristics of the emulsions and deposition parameters were determined. The increase in additive concentration (from 2 to 10 mL/L) in the emulsion resulted in poor adhesion of the coatings to the substrate (0B according to the ASTM D3359-23 standard). The coatings contained droplets of additives scattered relatively homogeneously in the chitosan matrix. The amount and dimension of the droplets were affected by the content of the additive in the emulsion. It was found that all of the additives had an impact on chitosan, as they formed new intermolecular hydrogen bonds. The roughness and wettability of the coatings were significantly affected by the concentration of additives in the dispersed systems. In most cases, increasing the concentration of additives led to lowering the wettability of coatings and increasing their roughness. Electrochemical studies confirmed that all of the investigated coatings improved resistance to corrosion compared to that of the bare titanium substrate. The coatings had different bactericidal factors with respect to the additive used. Mostly, they acted actively against *Staphylococcus aureus* compared to *Escherichia coli* bacteria. The microbicidal activity of the coatings was strongly related to the time of incubation of the bacteria strains. Of all additives, cinnamon bark oil and (cinnamaldehyde) acted the strongest against both pathogens. All coatings had a high cytotoxicity factor against both the MG-63 and FaDu cell lines. Nevertheless, they are very promising for applications that require a high antimicrobial performance.

## Graphical abstract

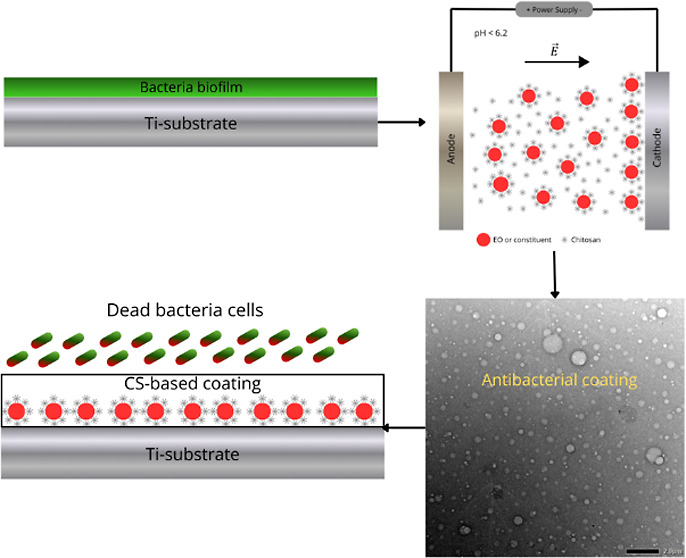

## Introduction

Electrophoretically deposited chitosan (CS)-based coatings are of interest as potential antimicrobial protection of metallic biomaterials.^1,2^ It is mainly due to the specific properties of chitosan. This chitin derivative, attained during its deacetylation or through extraction from fungi or crustaceans, is a biopolymer that occurs naturally.^3^ It has high wound-healing potential,^4^ strong antimicrobial response,^1^ low cytotoxicity,^5^ and very good resistance to electrochemical corrosion.^6^ Moreover, CS can form composites with various materials.^7^ Today, it is widely used as a matrix for more complex natural composites containing various substances of high microbicidal potential^8^ and wound-healing effect.^9^ One such substance is essential oils (EOs) and their components. It is widely known that some EOs have outstanding microbicidal and fungicidal properties.^10,11^ Therefore, they were examined as potential additions to the chitosan matrix. This approach is widely used in the food industry, where EO/chitosan coatings are commonly applied as protective layers against the decay of fruits and vegetables^12,13^ or to prolong the lifespan of meat or fish.^14,15^ However, similar coatings can also find use in biomedical engineering applications. There have been several works focused on the biological properties of such materials. Altiok et al.^16^ investigated thyme EO in the chitosan matrix as potential wound dressings. Their results suggested that such films may have applications in future wound-healing therapies. Similarly, Karami et al.^17^ obtained emulgel and xerogel films based on chitosan with *Thymus pubescens* EO as a promising wound treatment. They showed that such films not only acted strongly against different types of microbes but also had antioxidant abilities. It was another advantage of this approach, and such films may be used as potential wound dressings. Gaspar et al.^18^ added *Cymbopogon citratus* EOs to chitosan. They observed that a low concentration of EO in chitosan resulted in low cytotoxicity, and such films could be considered as a skin-treatment medium.

Among the EOs widely examined as potential additions to the CS matrix to improve its antimicrobial properties, several are very promising. Within them, EOs derived from cinnamon and thyme are considered suitable for biomedical applications.^19–21^ Cinnamon essential oils extracted from the bark (COB) or leaves (COL) of the cinnamon tree are divided according to the main constituents, cinnamaldehyde (CA) and eugenol (EU), respectively.^20,22,23^ In particular, COB are considered to be very microbicidal due to the presence of CA.^24,25^ CA is an oily yellowish liquid with a cinnamon-like smell that has low solubility in water but easily dissolves in ethanol.^26^ The antimicrobial potential of cinnamaldehyde is very strong in acting against, for example, *Escherichia coli*,^27^ *Candida albicans* and *Nakaseomyces glabrata*,^28^ *Mycobacterium tuberculosis*,^29^ and *Streptococcus mutans*.^30^ It was suggested^31^ that CA can alter the permeability of the *E. coli* and *Streptococcus aureus* bacteria membranes. The main component of COL, eugenol, is a phenolic aromatic substance with a pleasant smell, occurring mainly as a slightly yellow liquid of oily consistency.^32^ Its antimicrobial potential against human-associated microorganisms is directly related to the specific type of bacteria that it targets. Hemaiswarya and Doble^33^ reported that EU altered the permeability of the bacteria membranes of several Gram-negative bacteria, including *E. coli* *,* *Enterobacter aerogenes* *,* *Proteus vulgaris* *,* *Pseudomonas aeruginosa,* and *Salmonella typhimurium* *.* The antimicrobial effect on *E. coli* was also approved by Pei et al.^34^ Regarding Gram-positive bacteria, there is a consensus that EU acts weaker against them due to polysaccharide in their membranes, making permeability disruption much harder.^35,36^ Although experiments performed by Qiu et al.^37^ suggested that EU may be used as a base for future microbicidal treatments.

Thyme essential oil (TO) is extracted from *Thymus vulgaris* and *Thymus zygis* with concentrations of 37–55% thymol (TH) and 0.5–5.5% carvacrol (CAR).^19^ Its microbicidal potential is directly related to its chemical composition.^38^ EOs with a high content of phenolic monoterpenes, mostly TH, are considered to act stronger. According to Kryvtsova et al.,^38^ TO acted more strongly on Gram-positive bacteria than on Gram-negative strains. They also reported that the impact of TO on biofilms increased with increasing TO concentration. Regarding thymol, it is a crystalline substance that is easily soluble in alcohol but hardly in water.^19^ TH is a very strong microbicidal agent. It acted effectively against Gram-positive and Gram-negative bacteria.^39,40^ The mechanism of action is similar to those of the EU and CA and is based on the disruption of the membranes of bacteria.^19,39^ Furthermore, TH was found to be very useful in decreasing biofilm growth.^41^ As for carvacrol, it works very similarly to that of thymol. It also stops biofilm growth^41^ and disrupts the permeability of bacteria membranes.^42^ However, its impact on the microbicidal potential of TO is much weaker due to the very low concentration in the formula.

In our previous works^2,43^ we developed an electrophoretic deposition (EPD) route for the codeposition of CS with tea tree oil (TTO) and terpinen-4-ol. Robust and macroscopically homogeneous coatings were attained at a potential difference of 10 V during 5 min from the mixture consisting of 10 mL/L of EO and 2 g/L of CS in ethyl alcohol solution (50 vol % of EtOH). In the present work, the EPD route was adapted, and it mainly focused on the influence of the addition of various EOs and their constituents delivered from cinnamon (COB, COL, CA, and EU) and thyme (TO, TH, and CAR) to chitosan coatings on their adhesion strength, roughness, and surface properties, as well as electrochemical corrosion resistance, microbicidal properties, and cytotoxicity of coated titanium substrates.

## Materials and Methods

### Materials

COB, COL, CA, EU, TO, and CAR in the liquid state (product nos. W229105, W229210, W228613, E51791, W306509, and W224511, respectively), TH in the crystal state (no T0501), and medium molecular weight chitosan powder with a deacetylation degree of 75–85% (no 448877) was obtained from Merck, Poland. Titanium grade 1 sheets (0.5 mm thick) were bought from Shanghai Huaxia Industry Co. Ltd. (China). They were cut into rectangular samples with dimensions close to 15 mm × 36 mm and ground using 600-grit emery paper. Before the deposition process, they were washed with technical ethanol in an ultrasonic cleaner.

### EPD of Coatings

A CS (2 g/L) in a 0.5% acetic acid solution in water was utilized as the dispersed phase in the system developed for the deposition of coatings. A detailed procedure for obtaining such a solution was given elsewhere.^2,43^ The mixture was then stirred with pure ethanol in a volume ratio of 1:1. Finally, the appropriate amount of EO or its constituents was added to obtain dispersed systems with concentrations of 2, 6, or 8 mL/L (for COB, COL, TO, and CA) and 2 mL/L (for EU, TH, and CAR). All were subsequently blended using a Unidrive X1000D homogenizer (CAT, Germany) at 20,000 rpm for 6 min. The mixtures were later used for the deposition of the coatings. The electrophoretic light scattering technique was used to measure the zeta potential (*z*-potential) and conductivity depending on the pH of the obtained mixtures. Both parameters were determined utilizing a Zetasizer Nano ZS-90 from Malvern Instruments Ltd., UK. The pH measurements were conducted with an ELMETRON CPC-505 pH meter, Poland. Each measurement performed for the dispersed systems was repeated 3 times to achieve average results. Extraction of the *z*-potential from the obtained data was carried out using the Smoluchowski equation. The pH range in which measurements were made was set from 3 to 7 with a step of 0.5. To obtain each pH value, sodium hydroxide and hydrochloric acid were used. The systems applied for EPD were also investigated by transmission electron microscopy (TEM, JEOL JEM-ARM200F NEOARMex, Japan) at 60 and 200 kV. To prepare specimens for TEM investigation, a droplet of each mixture was settled on a carbon-coated copper grid and dried.

Selected area electron diffraction (SAED) was applied for phase analysis. The obtained SAED patterns were indexed by utilizing Java Electron Microscopy Software (JEMS, Pierre Stadelmann, Switzerland).

The EPD was performed in a cell equipped with two electrodes, where one was an AISI 316L stainless steel plate and the second was a Ti substrate. The electrodes were placed 1 cm apart. The process was conducted at a constant potential difference (10 V) of direct current (DC) obtained by an EX752 M multimode power supply unit (Aim-TTi, UK) for 5 min in each case. After the deposition, the obtained coatings were left to dry in still air.

### Adhesion Strength of Coatings

The adhesion strength of the obtained coatings to the ground titanium was measured by using the tape test according to the ASTM D3359-23 standard (method B). Orthogonal cross-cuts were performed by using an MFS 3000 multifunctional plate and an NT cutting knife (MTV Messtechnik, Germany). Then 1539M0025 transparent pressure-sensitive adhesive tape (ISO 2409) was stuck to the coating surface. After 90 s, the tape was removed from the surface, maintaining an angle close to 180°. Two tests were made for each coating. Scanning electron microscopy (SEM) FEI NOVA NanoSEM 450 and FEI Inspect S50 (The Netherlands) were utilized to analyze the sample surfaces after the tests. The class of adhesion was established based on visual aspects of the coating surface, including delaminations and defects around the cutting edges. The quantity of the coating removed from the substrate to the entire area was measured using Fiji.app software.^44^

### Microstructure, Surface Topography, and Surface Properties of Materials

Microstructural observations of the coated titanium were conducted employing the SEM described above on plan-view samples.

Structural characterization of the samples was conducted using a Vertex 70 V spectrometer (Bruker, USA) and Fourier transform infrared (FT-IR) spectroscopy. Pure CS coating as well as the CS coatings with the additives were analyzed using a Seagull attachment (Harrick Scientific, USA) by specular reflection technique. The measurements were performed in the range from 400 to 4000 cm^–1^ with a resolution of 4 cm^–1^ collecting 128 scans. OPUS 7.2 software (Bruker, USA) was used to process the obtained data.

The surface topography of the materials was acquired by utilizing a WYKO NT 9300 optical profilometer (Veeco, Bruker, USA). The roughness parameters, including Sa—arithmetic mean roughness, Sq—root-mean-square roughness, Rt—distance between the lowest valley and the highest peak of the measured area, and ISAD—image surface area difference, were extracted from data using Gwyddion software.^45^ Each measurement for each coating was repeated three times in areas of about 1.2 mm^2^.

The DSA25E system (Krüss, Germany) was applied for the analysis of wettability angles (WA) and surface free energy (SFE) with the sessile drop technique. As a polar liquid, distilled water was utilized, and methylene iodide was utilized as a nonpolar liquid. Nine measurements were performed for each sample and for each liquid. The WA values were gained by applying the ellipse-fitting method. The SFE values were extracted from the data using the Owens–Wendt–Rabel–Kaelble method and divided into polar and dispersive parts.

### Electrochemical Measurements

The stationary potential (OCP), polarization study (LSV), and electrochemical impedance spectroscopy (EIS) studies of the samples were carried out using an Autolab PGSTAT302N potentiostat equipped with an FRA32MEIS module and managed by Autolab NOVA 1.11 software (Metrohm Autolab B.V., The Netherlands). All tests were conducted in a Faraday cage to protect the electrochemical cell from electromagnetic noise. The reference was an Ag/AgCl electrode, and a Pt mesh was applied as the counter electrode. Hanks’ solution was utilized as the electrolyte. The OCP potential values were measured in deaerated solutions with pH = 7.4 at a temperature of 37 °C for 24 h. The polarization curves were carried out at a scan rate of 1 mV/s from −1.5 V to +2.5 V. Electrochemical impedance spectra were collected at the open circuit potential. The amplitude of the perturbation signal was 10 mV, and EIS spectra were plotted in the frequency range of 10^5^ Hz to 10^–3^ Hz.

### Evaluation of Antibacterial and Antibiofilm Properties

Bacterial strains *Staphylococcus aureus* ATCC 25923 and *E. coli* ATCC 25922 (American Type Culture Collection, USA) were used as marker reference strains in the study. Bacteria were cultured in Tryptic Soy Broth (TSB) (Becton Dickinson, USA) at 37 °C for 18 h. After incubation, cells were pelleted in the centrifuge (4000 rpm, 3 min) and subsequently rinsed three times with phosphate-buffered saline (PBS) (Chempur, Poland).

Following adjustment of the bacterial suspension to 10^6^ CFU/mL in PBS, 1 mL was applied to each well of a sterile 24-well plate (Nest Biotechnology, China), where the examined samples were placed. The plates were maintained at 37 °C for the 4 h incubation period. Upon completion of the incubation, the tested surfaces were washed with PBS to detach unbound bacterial cells.

Next, two procedures were performed on the tested samples:

- Bacterial viability was quantified by serially diluting the bacterial suspension from each evaluated well and culturing them on Mueller–Hinton agar (Thermo Fisher Scientific, USA). The plates were incubated at 37 °C for 24 h. Bacterial colonies were counted, and the result was converted to colony-forming units per milliliter (CFU/mL), based on the respective dilution of the bacterial suspension in each tested well. A reference glass surface was used as a control. Each experiment was repeated three times to ensure reliability of the results.^46^
- To assess biofilm formation,^47^ the tested surfaces were transferred to 1 mL of TSB followed by incubation at 37 °C for 24 h. Next, the samples were carefully rinsed with PBS, and subsequently, a new portion of TSB with 10% AlamarBlue (Sigma-Aldrich, USA) was applied. Incubation was continued for 20 min in the presence of AlamarBlue solution. Afterward, aliquots of 100 μL of supernatant from each well were pipetted into a black 96-well plate (Thermo Fisher Scientific, USA).

Fluorescence resulting from resazurin reduction was quantified with a SpectraMax Mini reader (Molecular Devices, USA) at λ_excitation_ = 544 nm and λ_emission_ = 590 nm. The reduction level was expressed as a percentage based on the following equation

$$\%\;resazurin\;reduction=\frac{S_{x}-S_{0}}{S_{100}-S_{0}}\cdot 100\%$$

*S*_*x*_—fluorescence intensity recorded for the examined sample; *S*_0_—fluorescence of the medium supplemented with 10% AlamarBlue reagent, serving as the 0% resazurin reduction reference (blank); and *S*_100_—fluorescence of the same medium with 10% AlamarBlue reagent after autoclaving at 121 °C for 15 min, corresponding to 100% resazurin reduction.

### Evaluation of Cytotoxicity of the Tested Samples

Cell viability was evaluated using MG-63 (ATCC CRL-1427) and FaDu (ATCC HTB-43) cell lines. Cells were incubated at 37 °C with 5% CO_2_ using Eagle’s Minimum Essential Medium (EMEM) supplemented with 10% fetal bovine serum (Gibco FBS) and ZellShield antimicrobial mixture (Minerva Biolabs, Germany). The cells were transferred to 24-well plates to obtain a density of 1.5 × 10^5^ cells per well. Test wells contained one of the studied titanium surfaces, while control wells served as cell-only references without any material present. The cells were exposed to the examined surfaces for 24 h under standard incubation conditions. Upon completion of the incubation, the nutritional medium was replaced by fresh culture medium supplemented with 10% AlamarBlue reagent (Sigma-Aldrich). The incubation process was prolonged for 4 additional hours at 37 °C to complete the assay. Further procedure was analogous to that described above (paragraph 2.6) in the case of bacteria.

## Results

### Deposition of the Coatings

To achieve stable dispersed systems containing cinnamon EOs, thyme essential oil, and their constituents for subsequent achievement of robust coatings, the impact of acidity on the *z*-potential and conductivity was measured. The data obtained are shown in Figure 1a–f. Almost all of the mixtures analyzed that contained chitosan and specific EO or constituent revealed comparable values of the mentioned electrokinetic properties with only slight differences. In the case of COB and CA (Figure 1a,b), *z*-potential and conductivity were measured for three different concentrations of the specific substance in the dispersed system. The increase in the concentration affected only a small amount of both parameters, slightly decreasing the values of the *z*-potential. For mixtures with COL and EU (Figure 1c,d), there were differences in the pH range of 5.0–6.5, where the *z*-potential obtained for the mixture with EU was much lower than that for the mixture with COL. The most visible differences were observed for systems involving TO and its constituents (Figure 1e,f). *Z*-potential of the system with CAR was the lowest of the three compounds in the range of pH from 3.0 to 5.0. The most stable decrease in value was observed for the TO-containing mixture over the whole range analyzed. For the system with TH, the *z*-potential values were very irregular. There was a very rapid and significant drop in the value between pH 4.5 and 5.0. This irregularity may be related to the fact that TH was added to the dispersed system in a solid form (powder).

**Figure 1:**
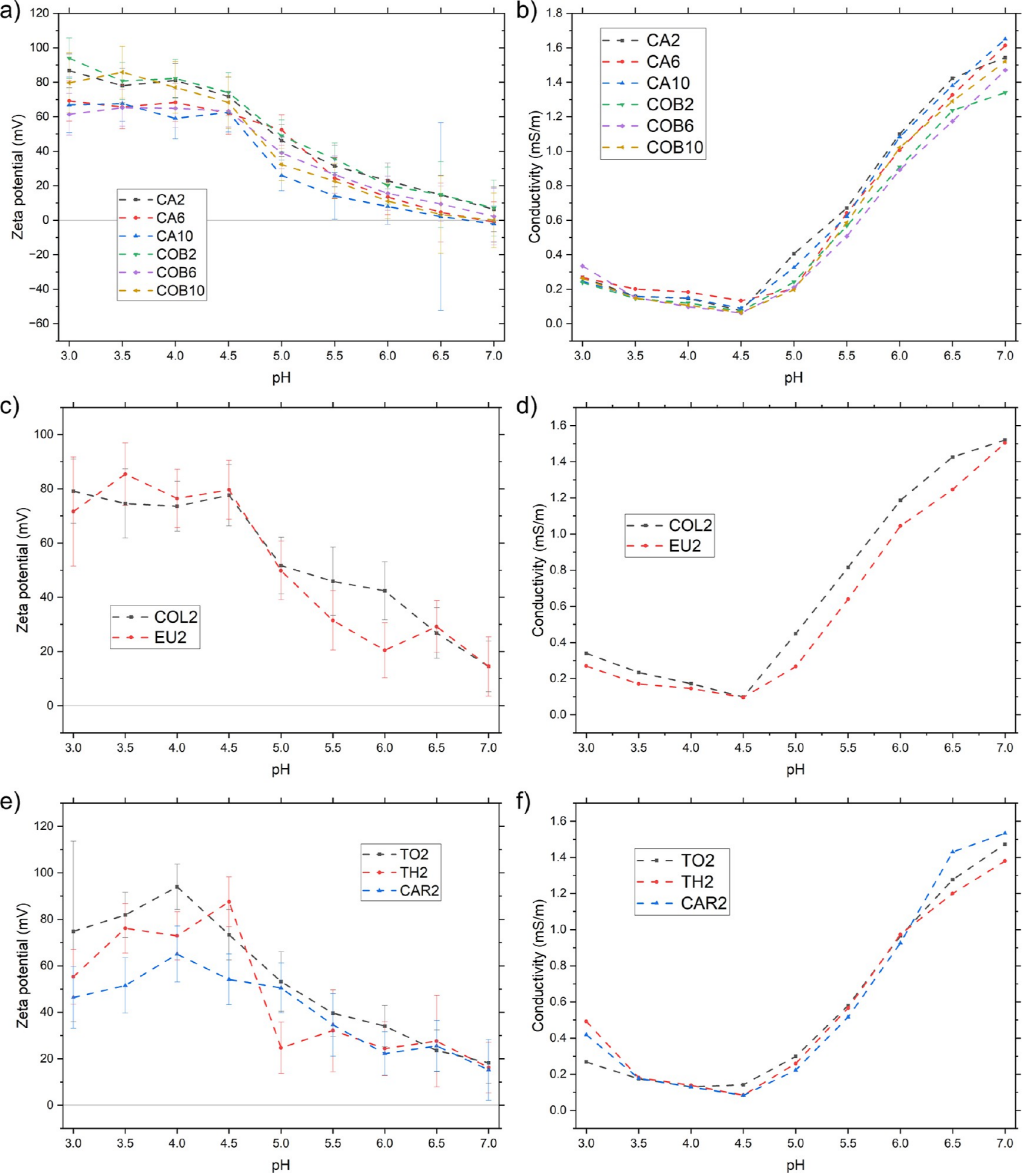
*Z*-potential (a,c,e) and conductivity (b,d,f) of the mixtures with COB and CA (a,b), COL and EU (c,d), and TO, TH, and CAR (e,f). The number in the system designation indicates the concentration in mL/L.

For all of the analyzed mixtures, the highest *z*-potential was achieved for a narrow pH range of 3.5 to 4.5. No isoelectric point was detected in the measured pH range for any mixture. The minimal conductivity level was measured for a pH close to 4.5 in each case. In fact, each mixture analyzed had an original pH very close to 4.5, suggesting that EOs and the constituents had a very low impact on the pH of the mixtures. Information on the pH value of the dispersed system is very important because CS in dispersions with a pH over 6.3 becomes deprotonated, and the EPD is not possible.^48^

The dispersed systems described above were investigated using TEM. In all of the systems analyzed, the compounds added to the CS solution were visible as droplets (Figure 2a–g) scattered regularly in the matrix, forming a continuous film. The diameter of the droplets ranged from less than 50 nm to 1 μm. All antibacterial additives analyzed, except CA, revealed an amorphous nature, confirmed by the presence of a diffuse halo in SAED patterns. In the case of CA, nanocrystalline precipitates were observed to create dendritic-like configurations in CS (Figure 3a). Their size was estimated to be in the range of 60 to 200 nm (Figure 3b). These observations suggest that interaction between the CA and the CS solution mixed with ethyl alcohol causes some CA to form crystals during air drying of the sample, while some remains as droplets. The crystalline nature of the precipitates was confirmed by selected area electron diffraction (Figure 3c). The analysis of SAED patterns revealed that diffraction spots are mostly covered with crystallographic data for CA (monoclinic), but there are also spots that could not be associated with CA. It is assumed that during crystallization, CA can form complexes with chitosan and that the corresponding diffraction spots may originate from those compounds.

**Figure 2:**
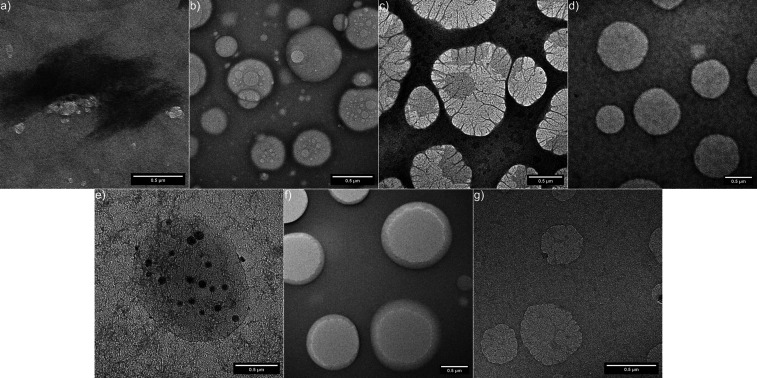
TEM images of the mixtures involving 2 mL/L of (a) COB, (b) CA, (c) COL, (d) EU, (e) TO, (f) TH, and (g) CAR.

**Figure 3:**
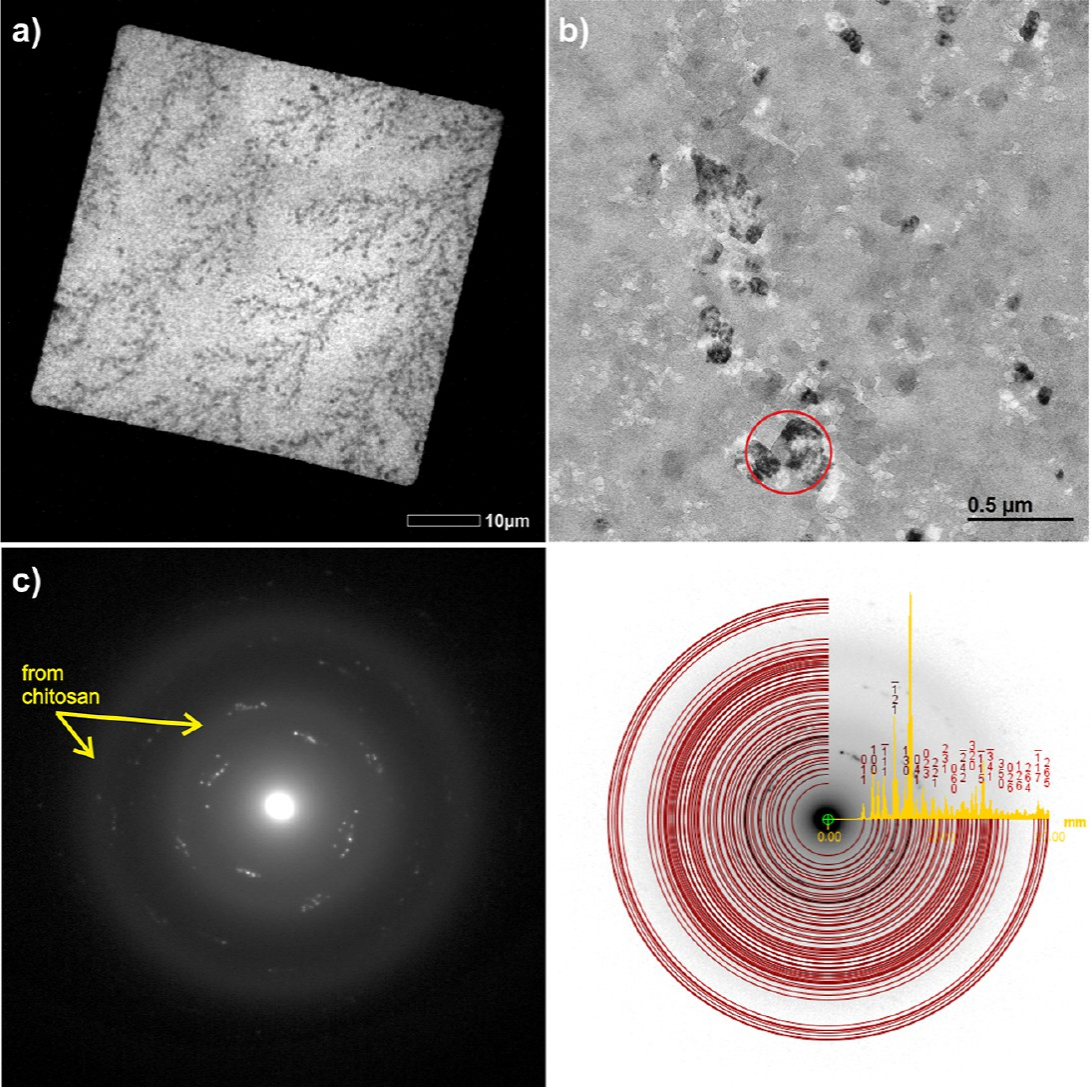
TEM images of the dispersed systems containing CA: (a) crystals with dendritic-like forms visible at low magnification, (b) crystalline precipitates, and (c) SAED pattern from the crystals designated with a ring in Figure 3b with identification as CA (monoclinic). The diffuse halo from chitosan is marked with yellow arrows in Figure 3c.

It was also found that only in the case of CA and COB were the small droplets embedded in the large ones (Figure 2a,b), indicating the coagulation of both EO and its constituent. This is a result of the presence of CA in an undissolved state in the dispersed systems. It was also observed that for COL and EU (Figure 2c,d), as well as for TO, TH, and CAR (Figure 2e,f,g), the droplets did not have a circular shape but were more or less irregular with ragged edges. The source of this phenomenon might also be attributed to the interactions between these compounds and CS, especially the formation of bonds between them.

The schematic diagram of the EPD process of codeposition of chitosan molecules with EO or its pure constituent is shown in Figure 4. During coating deposition, the changes in current density over time were monitored (Figure 5a–c). It was observed that the EOs and the constituents had an impact on the deposition process. For systems with COB and CA, the shapes of the recorded curves are very similar to each other, with a subtle but noticeable tendency for current density to decrease over time across the entire measured range (Figure 5a). It was also observed that the concentration of the addition to the dispersed system influenced the current density values. The significant changes were observed for the mixtures with 6 mL/L of COB or CA, then 2 mL/L, and finally 10 mL/L. There were also several minor fluctuations noticed in the current density values, mostly for the systems containing 2 mL/L of both compounds. For the systems with COL and EU, the shapes of the curves were also similar to each other, with more fluctuations for the system containing COL (Figure 5b). For that system, the current deposition values were higher than those for the system with EU. The most visible differences in the curve shape were observed for TO and its constituents (Figure 5c). The highest values of current density were observed for the system with TH. The curves registered for the systems with TO and CAR had many differences. In the case of the system with CAR, the values of current density initially increased, then stabilized at a similar level, and finally gradually decreased. The reverse behavior was observed for the TO-containing system. First, it decreased, then remained stable, and finally increased. Such fluctuations and changes may be related to water electrolysis leading to the formation of gas bubbles, which were visible in the mixture during the deposition process.

**Figure 4:**
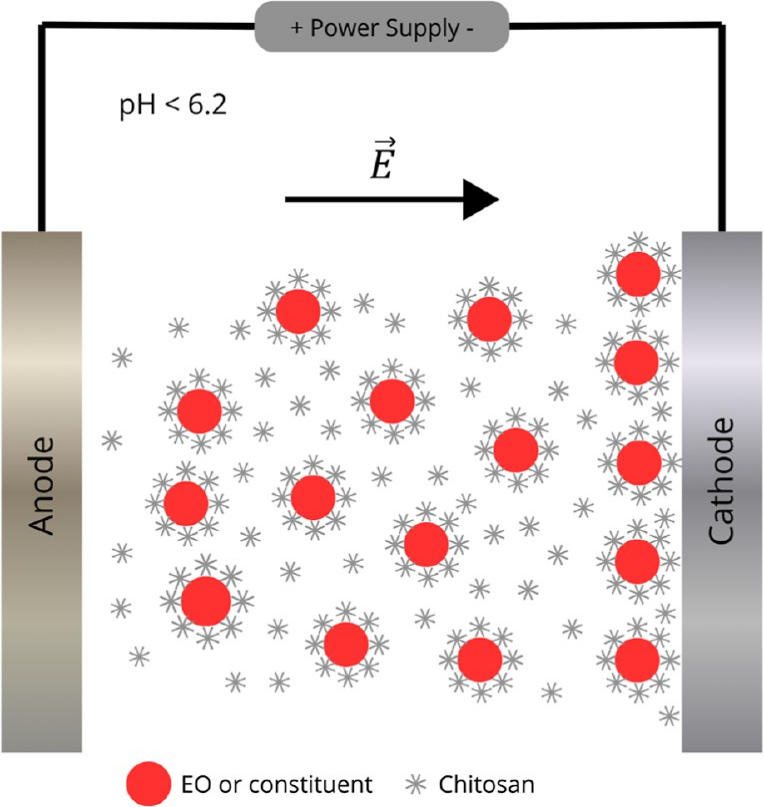
Scheme of the EPD of chitosan-based coatings containing EO or its pure constituent.

**Figure 5:**
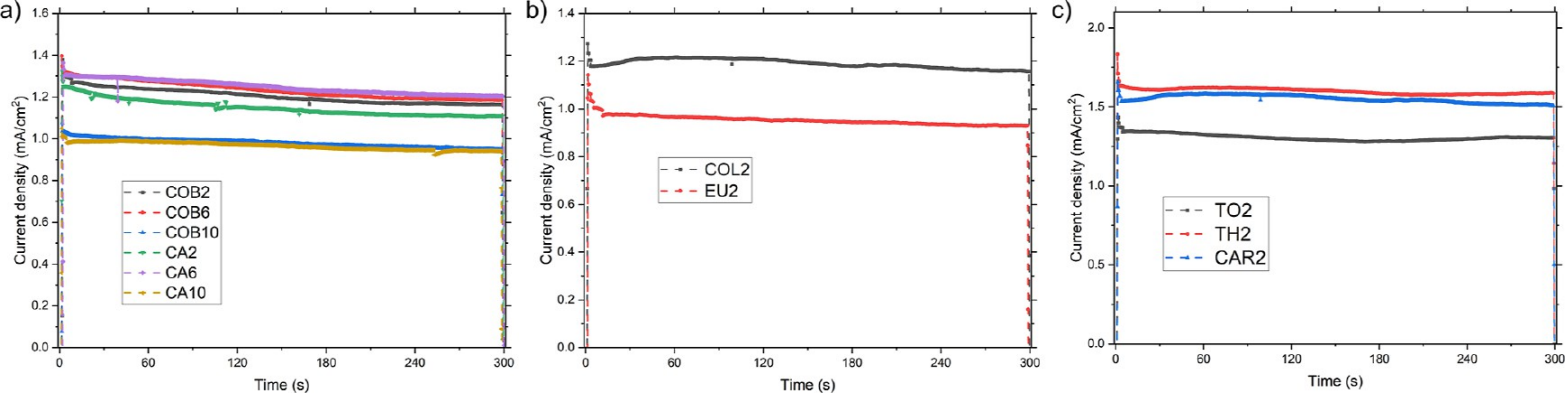
Current density during deposition of the CS-based coatings from the dispersed systems with (a) CA and COB, (b) COL and EU, and (c) TO, TH, and CAR according to the time of the EPD.

The coatings obtained from each dispersed system were visually very similar to each other. They were smooth, with very low traces of bubbles from electrolysis on the edges of the samples. Even changes in the concentration of EO or the constituent did not greatly affect the visual appearance of the coatings. Only some kind of haziness and yellowness, due to the color of the additions, was visible in the coatings containing 6 or 10 mL/L of COB or CA.

### Influence of Antimicrobial Agents on Adhesion Strength and Homogeneity of the Coatings

First, the chitosan coatings incorporating COB, COL, and TO were deposited from the systems containing 2 or 6 or 10 mL/L of EO. In the case of the COB-containing coatings, the adhesion strength was found to be high, of class 4B, for each concentration of EO in the dispersion system (Figure 6a–c). There were only minor delaminations on cuts and cross sections, mostly visible for the coatings obtained from the mixtures with the lowest COB concentration.

**Figure 6:**
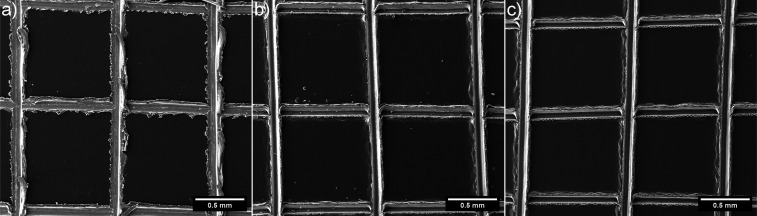
SEM images of the surface of the COB/CS-coated titanium after the tape tests. The coatings were deposited from the mixtures with 2 mL/L (a), 6 mL/L (b), and 10 mL/L (c) of COB.

For the coatings with COL and TO (Figures 7a–c and 8a–c, respectively), concentrations greater than 2 mL/L in the mixtures utilized for the deposition contributed to the poor adhesion strength, which was class 0B in each case. In contrast, the coatings achieved from the mixtures of 2 mL/L of EO adhered well to the substrate, and adhesion class 4B was determined. Only slight detachments in the vicinity of the incisions were found.

**Figure 7:**
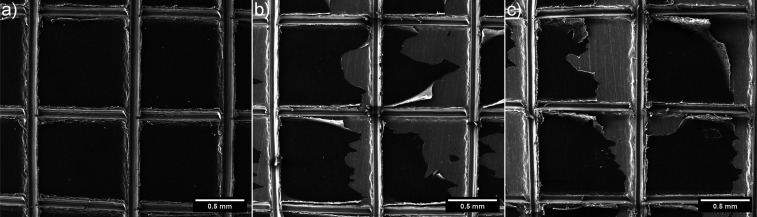
Surface of the COL/CS-coated titanium after the adhesion tests. The coatings were deposited from the mixtures with 2 mL/L (a), 6 mL/L (b), and 10 mL/L (c) of COL. SEM images.

**Figure 8:**
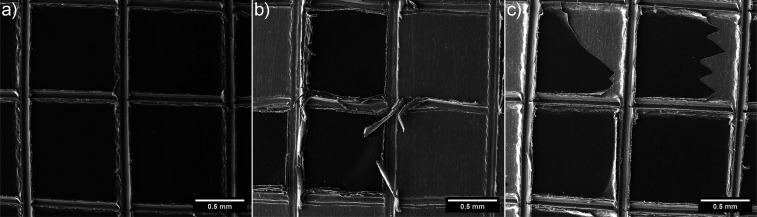
Surface of TO/CS-coated titanium after the adhesion tests. The coatings were achieved from the mixtures with 2 mL/L (a), 6 mL/L (b), and 10 mL/L (c) of TO. SEM images.

The acquired data on the adherence of the coatings to the substrates, along with information about the composition of EOs, allowed us to choose appropriate concentrations of the constituents in the mixtures utilized for the deposition process. For CA, which is the main component of COB, the coatings were deposited for the same concentrations as for EO. It was observed that, similarly to the COB/CS coating, the adhesion strength for each analyzed CA/CS coating was 4B (Figure 9a–c). There were only minor delaminations visible on the cutting edges. For the remaining constituents, a content of 2 mL/L was selected mainly because of the adhesion strength obtained for the coating with the respective EOs. For each coating containing the EOs components, the adhesion class of 4B was detected with a slight detachment of small coating areas in the vicinity of incisions (Figure 10a–c). It was found that for the coatings incorporating TO, TH, and CAR, visible delaminations were slightly greater than those for the other coatings (Figure 10b,c). This may suggest that for higher concentrations of antimicrobial agents in the initial mixtures utilized for the deposition, the coatings would be completely removable after the tape test.

**Figure 9:**
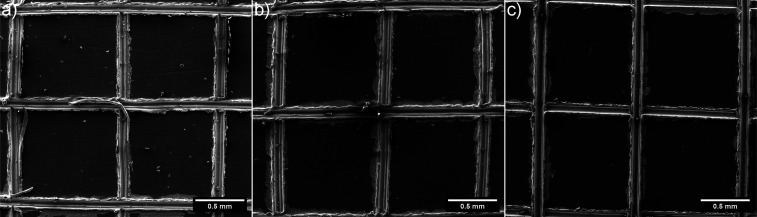
Surface of the CA/CS-coated titanium after tape tests. The coatings were deposited from the mixtures involving 2 mL/L (a), 6 mL/L (b), and 10 mL/L (c) of CA. SEM images.

**Figure 10:**
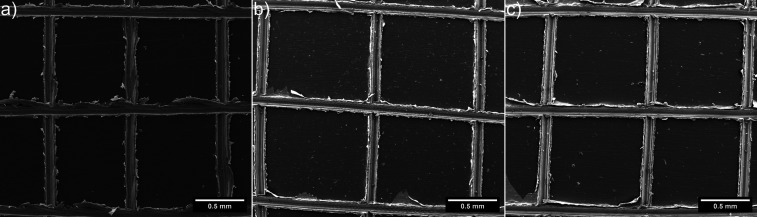
Surface of the EU/CS (a), TH/CS (b), and CAR/CS (c) coated titanium after the tape tests. The content of the constituent in the system was 2 mL/L. SEM images.

The adhesion strength tests showed that there were significant differences for the coatings incorporating various additives, COB, CA, COL, TO, EU, TH, and CAR. These differences may be related to the chemical structures of all the substances analyzed. COB and its main component CA are supposed to create an imine bond between CA molecules and chitosan (a double carbon–nitrogen bond C=N) in an acidic environment.^49–52^ This directly affects the bond formation between molecules from the dispersive system and the metallic substrate. These complexes create physical and chemical bonds between their molecules and the metallic surface.^52^ For COL and its main component, EU, which belongs to the phenylpropene group, the interaction between EU and chitosan is very similar to that of CA. EU also creates a bond between the nitrogen atom in chitosan and the carbon atom in EU, but it is not a double bond but a single bond (Michael’s type adduct).^53,54^ The interactions of such complexes with the metallic surface may be very similar to those of CA. Differences in the adhesion strength may be related to weaker interactions. Regarding TO and its constituents TH and CAR, both TH and CAR are phenols, and a natural monoterpene derivative of cymene, and CAR is an isomer of TH. Their interactions with chitosan are also based on the creation of bonds between nitrogen atoms and carbon atoms in both molecules.^55,56^ Similarly, adhesion weakness may also be related to the strength of these bonds.

Comparing obtained results with results of our previous works on chitosan coatings with tee tree oil (TTO) and terpinen-4-ol^2,43^ as well as citronella oil (CEO) and citronellol,^57^ it can be clearly seen that the addition of EO or its constituent affects the adhesion strength of coatings. We proved that the addition of EO or a constituent to the chitosan coating worsened the adhesion strength with an increase in concentration in the dispersed system used for deposition, but it varies depending on the type of EO. For example, we showed that the adhesion of TTO/chitosan coatings worsened for those deposited from systems of oil concentration greater than 10 mL/L. For coatings obtained from systems below this level of concentration, the adhesion class of coatings was very high. However, for CEO/chitosan coatings, we observed that only coatings obtained from systems of concentrations 1 and 2 mL/L exhibit high adhesion to the substrate. Above 2 mL/L oil concentration in the system, the adhesion of coatings worsened to complete delamination for the coatings obtained from a system of 10 mL/L concentration. Similarly, in this study, we also observed that different EOs affected the adhesion strength of the coatings differently. In the case of COB, we showed that the adhesion strength was high for all analyzed coatings relative to the concentrations in the dispersed systems used for EPD. On the other hand, the coatings obtained from the systems containing COL and TO exhibited high adhesion only for those deposited from the systems of 2 mL/L oil concentration. Above that level, the coatings were easily removable from the substrate.

The investigation of the morphology of the coatings revealed that despite the initial form of EOs and their constituents, they appeared on the surface of the coatings as droplets with diameters up to 20 μm. In most cases, especially for COB and CA, these droplets created chain-like arrangements that were aligned according to the direction of grinding of the substrates (Figures 11 and 12, respectively). It was also found that despite the increase in their concentration in the mixture used for EPD, the number of visible droplets did not increase significantly, which may be attributed to the solubility of CA in ethyl alcohol.^26^

**Figure 11:**
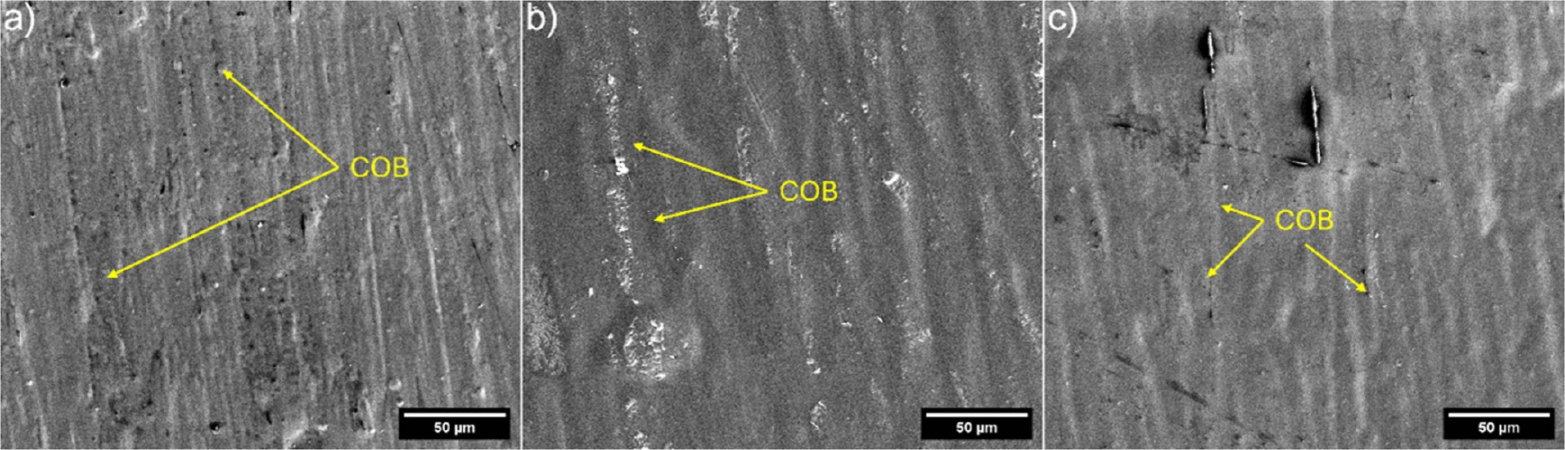
Surface of the COB/CS coatings achieved from the mixtures containing various COB content: (a) 2 mL/L, (b) 6 mL/L, and (c) 10 mL/L, SEM.

**Figure 12:**
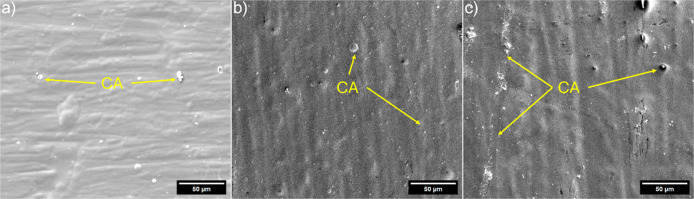
Surface of the CA/CS coatings achieved from the mixtures containing various CA content: (a) 2 mL/L, (b) 6 mL/L, and (c) 10 mL/L, SEM.

Similarly, as for COB and CA droplets, droplets of COL and EU also create chain-like forms on the surface of the coatings, which were oriented along the direction of the grinding substrates (Figure 13a,b, respectively). They were more visible in the case of COL droplets than in the case of COB, despite the high solubility of eugenol in ethyl alcohol.^58^ This may indicate that other constituents of COL affect its solubility in different media and allow for the creation of such droplets in the CS matrix. Consequently, in the CS coatings with TO and its constituents, both TO and TH also formed very small and well-scattered droplets across the surface of the coatings (Figure 13c,d). It is a result of the high solubility of TH in ethyl alcohol.^59^ For CAR, there were more visible droplets that create chain-like forms (Figure 13e), identical to that described above. This is due to the low solubility of carvacrol in a chitosan-ethyl alcohol solution.^60^

**Figure 13:**
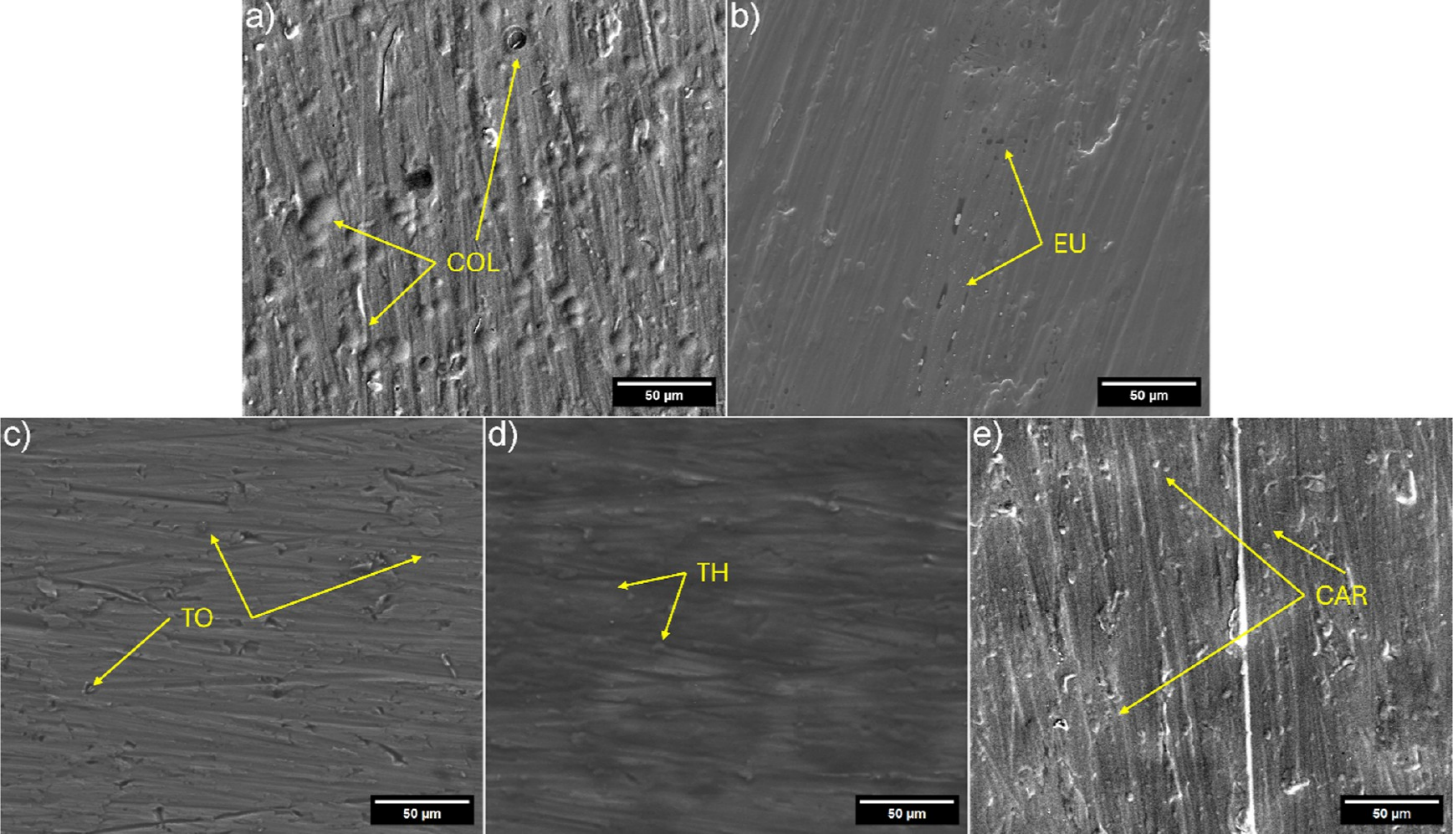
Surface of the CS-based coatings achieved from mixtures containing the following constituents (concentration 2 mL/L): (a) COL, (b) EU, (c) TO, (d) TH, and (e) CAR, SEM.

The FT-IR spectra of the CS-based composite coatings (Figure 14b–e) reveal bands characteristic not only of CS but also of specific additives. Structural analysis of both chitosan and individual additives by FT-IR spectroscopy has been presented in detail in our previous publications.^61^ As CA is the major constituent of COB, the corresponding spectra for COB/CS coatings (Figure 14b,d) are similar to each other. An analogous situation exists for EU/CS and COL/CS and the respective spectra (Figure 14c,e). In the spectra for the composite CS-based coatings, shifts of some bands to lower wavelengths are observed, which indicates the development of new intermolecular interactions between specific molecules and CS chains. The band at 1085 cm^–1^ for the C–O–C stretching in the six-membered heterocyclic rings of chitosan changes its position after the introduction of cinnamon oil leaf type and eugenol to 1068 cm^–1^ and 1067 cm^–1^, respectively. This could be explained by the formation of hydrogen bonds between the oxygen atoms in the rings and the hydroxyl groups of EU molecules (Figure 15a). As EU is the basic component of COL, the observed shifts in the spectra of CS coatings with these additives are similar. In the spectra of the CS coatings with COB and CA, a potential shift cannot be observed, as the analyzed band is overlapped by the bands attributed to the corresponding additives. However, in the structure of CA, which is also the main component of COB, there are no functional groups capable of forming intermolecular interactions with oxygen atoms in the rings of CS chains. The position of the band at 1032 cm^–1^ for the C–O–C stretching in the β-1,4-linkages between the rings is not changed in the spectra for the CS coatings with all additives, which indicates that their oxygen atoms are not involved in the development of new intermolecular interactions. This is due to steric hindrance connected with the occurrence of substituted rings in their vicinity, which prevents certain molecules from approaching. The band at 1420 cm^–1^ for the C–H stretching in –CH_2_OH moieties is shifted to 1412 cm^–1^, 1412 cm^–1^, 1411 cm^–1^, and 1418 cm^–1^, respectively, for the four analyzed coatings of chitosan with the additives. This is due to the formation of hydrogen bonds between these hydroxyl groups and oxygen atoms in methoxy groups of eugenol molecules or aldehyde groups of cinnamaldehyde molecules (Figure 15b,d). Since these hydroxyl groups are attached to the rings through methylene groups, they are more exposed and more capable of forming new hydrogen bonds than hydroxyl groups bonded directly to the rings. A significant change is the shift of the band at 1657 cm^–1^ for the C=O stretching in *N*-acetyl substituted amino groups to 1642 cm^–1^ and 1638 cm^–1^ for the CS coatings with COL and EU. Thus, strong hydrogen bonds are formed between oxygen atoms in carbonyl groups and hydroxyl groups in EU (Figure 15c). With the addition of COB and CA the band at 1323 cm^–1^ is shifted to 1318 cm^–1^ and 1315 cm^–1^, respectively, while after the introduction of the other two additives, the shift is not observed. This suggests the formation of hydrogen bonds between amino groups in the chitosan chains and oxygen atoms in the aldehyde groups of cinnamaldehyde (Figure 15e). The aldehyde group in the molecule of this additive is not attached directly to the aromatic ring but through an unsaturated hydrocarbon chain. Thus, CA molecules could approach amine groups bonded directly to the rings, despite the steric hindrance associated with the presence of adjacent substituents in the CS chains. Based on this, it can be assumed that a similar mechanism for the formation of new intermolecular interactions involves CA molecules and hydroxyl groups bonded directly to the rings, although no corresponding bands can be distinguished to confirm this explicitly (Figure 15f). For EU molecules, methoxy groups with oxygen atoms that could potentially form the same type of hydrogen bonds are attached directly to aromatic rings, limiting their ability to approach amino and hydroxyl groups bonded to the rings. Therefore, the shifts of analogous bands for the COL/CS and EU/SC coatings are not observed. Finally, the band at 1569 cm^–1^ for the C–N stretching changes its position after the introduction of the four additives to 1560 cm^–1^, 1564 cm^–1^, 1561 cm^–1^, and 1563 cm^–1^, respectively. The change in the position of this band for the CS coatings with different additives originates from two different mechanisms. For the COL/CS and EU/SC coatings, this effect is related to the previously described interactions between hydroxyl groups in EU and carbonyl groups of *N*-acetyl substituted amino groups in the CS chains (Figure 15f). As the atoms of C–N bonds are not directly involved in their formation, the observed shift is relatively small. In contrast, for the COB/CS and CA/CS coatings, it is associated with the mentioned interactions between aldehyde groups in CA molecules and amino groups in the CS chains (Figure 15b).

**Figure 14:**
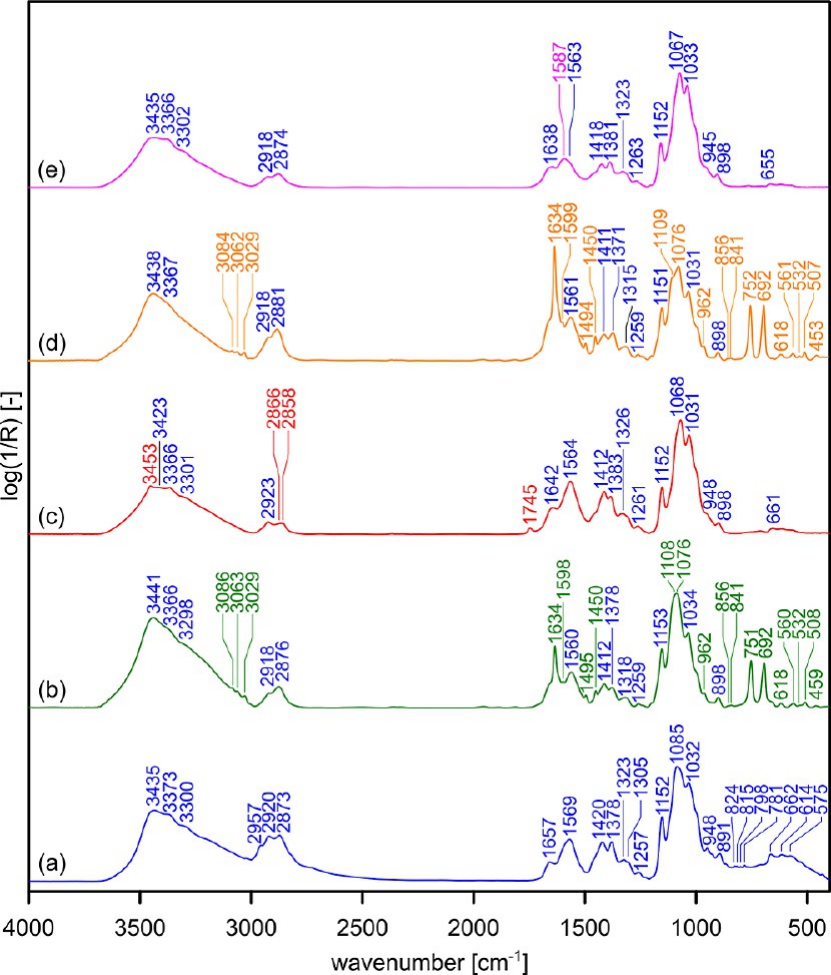
FT-IR spectra of the CS (a), COB/CS (b), COL/CS (c), CA/CS (d), and EU/CS (e) coatings. The bands for CS are marked with blue, for COB with green, for COL with red, for CA with orange, and for EU with pink color.

**Figure 15:**
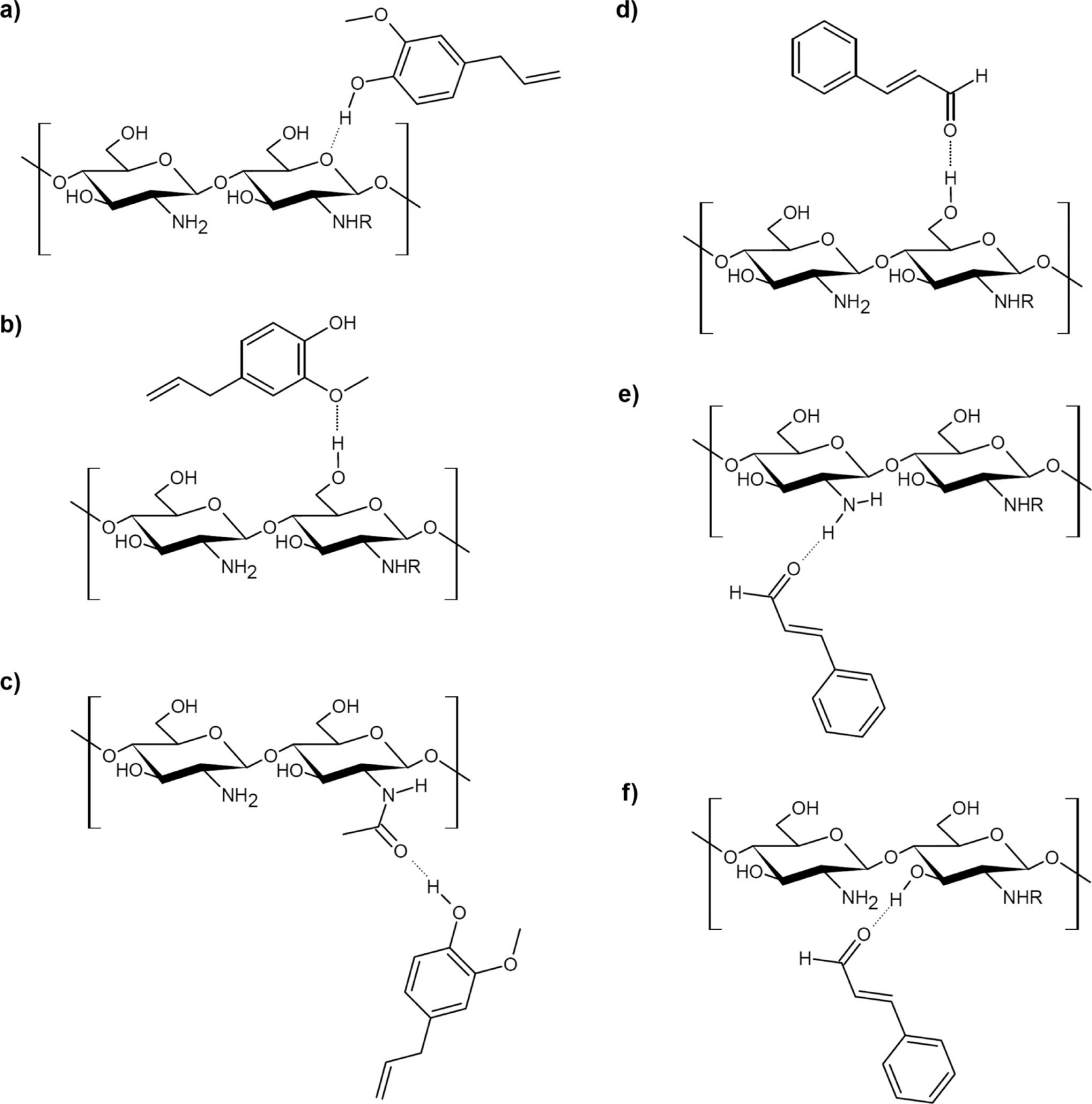
Proposed mechanisms of the interactions formed between EU molecules and the CS chains in the COL/CS and EU/CS coatings involving oxygen atoms in the chitosan rings (a), hydroxyl groups in the –CH_2_OH moieties (b), *N*-acetyl substituted amino groups (c), as well as between CA molecules and the CS chains in the COB/CS and CA/CS coatings involving hydroxyl groups in the –CH_2_OH moieties (d), unsubstituted amino groups (e), and hydroxyl groups attached directly to the rings (f) (–NHR is *N*-acetyl substituted amino group).

FT-IR spectra of the CS coating and the CS-based composite are shown in Figure 16, while their in-depth data are collected in Table S2.

**Figure 16:**
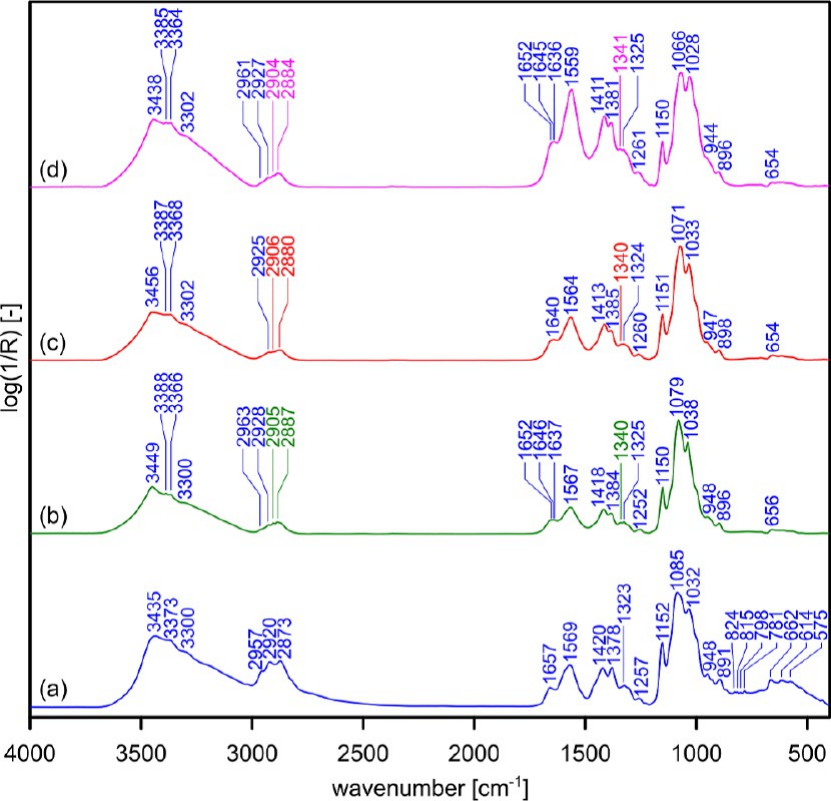
FT-IR spectra of CS (a), TO/CS (b), CAR/CS (c), and TH/CS (d) coatings. The bands for CS are marked with blue, for TO with green, for CAR with red, and for TH with pink.

The spectra of the TO/CS, TH/CS, and CAR/CS coatings (Figure 16b–d) reveal a few additional bands attributed to pure components. The main component of thyme oil is thymol; hence, the spectra for the CS-based composite coatings are similar to each other. CAR is the structural isomer of TH, from which it differs in the position of the hydroxyl substituent on an aromatic ring. Therefore, the spectrum for the CAR/CS coating is similar to those of the others. The observed shifts of some bands toward lower wavenumbers indicate the development of new intermolecular interactions between the molecules and the CS chains. The band at 1085 cm^–1^ for the C–O–C stretching in the chitosan rings is shifted to 1079 cm^–1^, 1071 cm^–1^, and 1067 cm^–1^ after the addition of TO, CAR, and TH, respectively. The mechanism of the associated interactions is the same as for the EU/CS coatings; that is, hydrogen bonds are formed between oxygen atoms in the rings and hydroxyl groups of CAR or TH molecules (Figure 17a). The band at 1657 cm^–1^ for the C=O stretching shows unusual changes in the position. After the introduction of both TO and TH, a set of three overlapping but distinguishable bands appear instead, at 1652 cm^–1^, 1646 cm^–1^, and 1637 cm^–1^, as well as at 1652 cm^–1^, 1645 cm^–1^, and 1636 cm^–1^, respectively. The introduction of CAR results in a typical shift of 1640 cm^–1^. The occurrence of three bands with similar positions suggests that TH molecules form three types of hydrogen bonding arrangements involving *N*-acetyl substituted amino groups, thus providing the formation of three different types of ordering of the CS chains. We previously described a similar mechanism for the CS coatings enriched with citronellol and geraniol.^57^ It can be assumed that this effect is related to the characteristic physical properties of TH, which in its pure form is not a liquid like CAR but a solid with a certain solubility (Figure 17b). The introduction of the three additives results in the shift of two bands at 1569 cm^–1^ and 662 cm^–1^ for the N–H bending to 1567 cm^–1^, 1564 cm^–1^, and 1559 cm^–1^ as well as 656 cm^–1^, 654 cm^–1^, and 654 cm^–1^, respectively. This indicates the formation of typical hydrogen bonds between the hydroxyl groups of CAR and TH molecules and the amino groups in the CS chains (Figure 17c). In the structure of both additives, the hydroxyl group is bonded directly to aromatic rings with other substituents in the vicinity. However, the observed changes in the spectra indicate that this does not lead to steric hindrance preventing the formation of intermolecular interactions. Therefore, the molecules can acquire the appropriate spatial orientation relative to the CS chains to form new hydrogen bonds. Finally, the band at 1420 cm^–1^ for the O–H stretching is shifted to 1418 cm^–1^, 1413 cm^–1^, and 1411 cm^–1^, which also reveals the formation of hydrogen bonds between hydroxyl groups of CAR and TH molecules and the CS chains (Figure 17d). Considering the formation of new intermolecular interactions with both amine groups attached to the CS rings and hydroxyl groups in the –CH_2_OH moieties, it can be considered that new hydrogen bonds are also formed with the involvement of hydroxyl groups bonded directly to the CS rings (Figure 17e).

**Figure 17:**
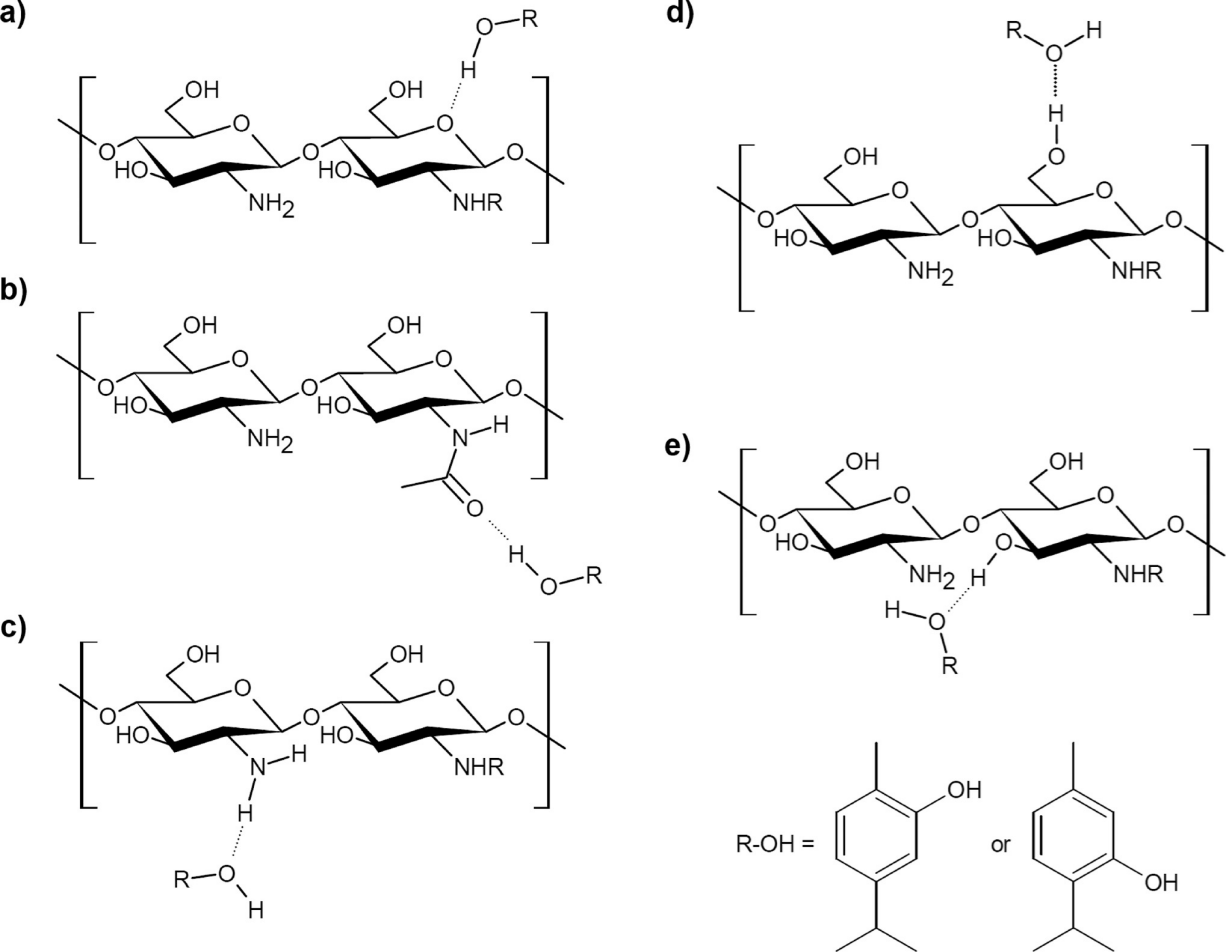
Proposed mechanisms of the interactions formed between CAR or TH molecules and the CS chains in the TO/CS, CAR/CS and TH/CS coatings involving oxygen atoms in the chitosan rings (a), *N*-acetyl substituted amino groups (b), unsubstituted amino groups (c), hydroxyl groups in the –CH_2_OH moieties (d), hydroxyl groups attached directly to the rings, and (e) (–NHR is *N*-acetyl substituted amino group).

### Surface Properties

The roughness analysis showed that the incorporation of EOs and their constituents in CS, except EU, increased the roughness parameters and ISAD compared to those of the pure CS coating (Figure 18a,b). It was also detected that their most significant changes were obtained for the coatings containing COB and CA. Moreover, the higher the concentration in the initial dispersion system, the higher were the roughness parameters and ISAD. For the coatings achieved from the systems including 10 mL/L of COB or CA, the ISAD was greater than 80%, and the roughness parameters, *S*a and *S*q, were greater than 2 μm. Comparing the data obtained with the roughness parameters and ISAD for the uncoated titanium substrate reported in our previous work,^2^ it was found that all analyzed coatings, except the EU/CS, had each parameter higher than the substrate.

**Figure 18:**
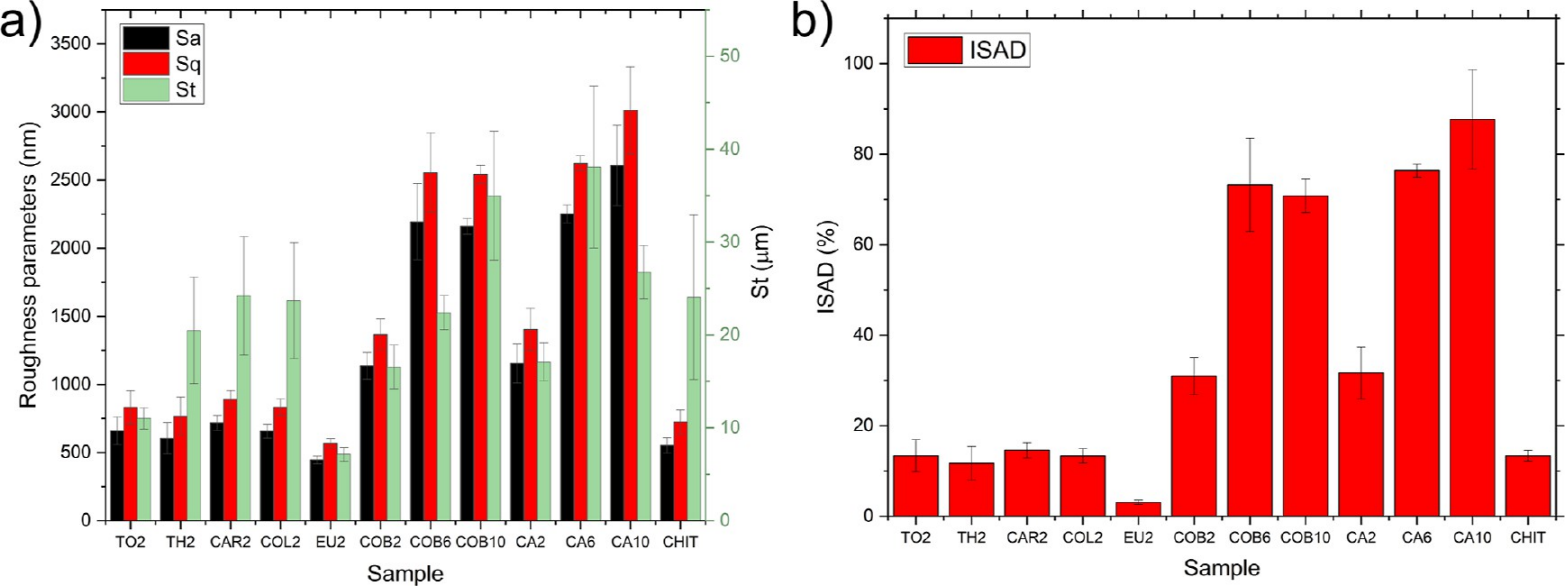
Surface metrology parameters including roughness (a) and ISAD (b) of the CS and CS-based coatings on titanium. The coatings are designated by their oily component and concentration (mL/L) in the mixture used for EPD.

The results of the WA and SFE measurements are listed in Figure 19a,b. The addition of EOs and the constituents decreased the WA values of the coatings for both water and diiodomethane (Figure 19a). It was also found that the higher the amount of oil in the mixture used for EPD, the lower the WA values of the coatings for both water and diiodomethane. Only for TO, COL, and COB at the concentrations of 2 mL/L, the WA values of the coatings for water were close to and higher than 90°. The coatings had a higher SFE with a more visible polar part than the pure chitosan coatings (Figure 19b). In general, the disperse component contributed mainly to their SFE value. It was also observed that the coatings containing higher concentrations of CA and COB and the coatings containing TH and CAR had a more noticeable polar part than those of the rest.

**Figure 19:**
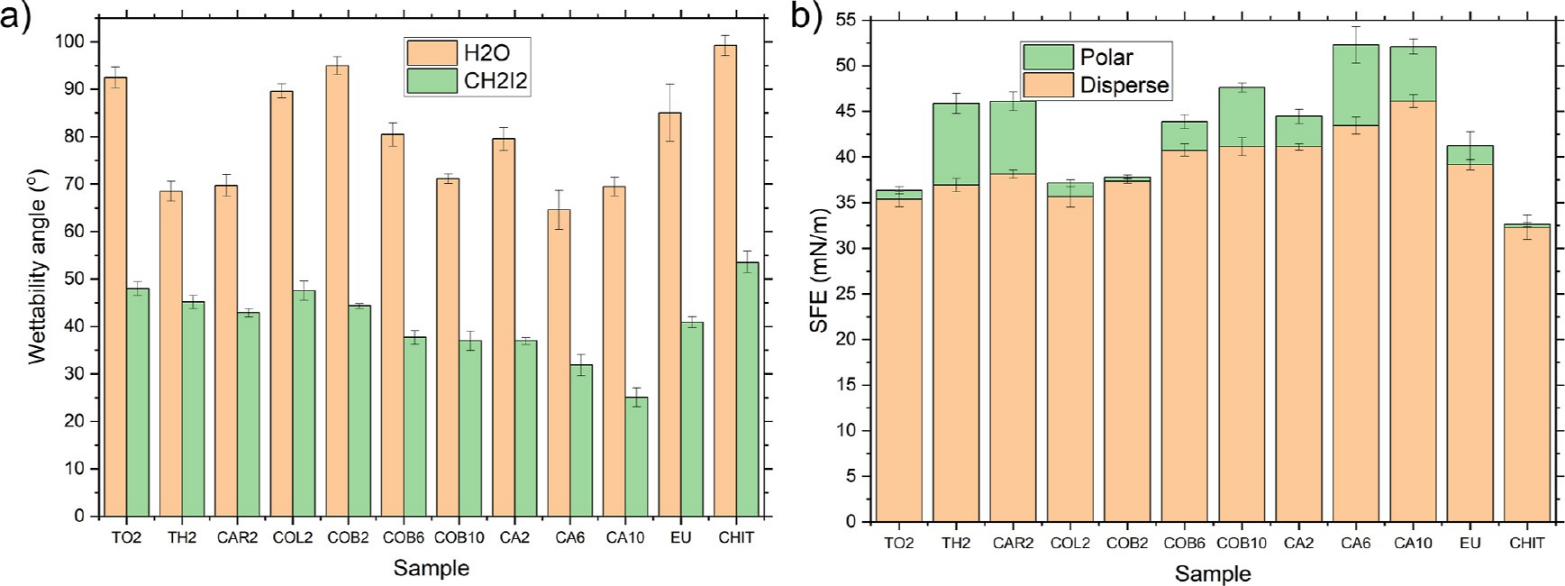
WA (a) and SFE (b) of the pure CS and CS-based composite coatings. The coatings are designated by their oily component and concentration (mL/L) in the mixture used for EPD.

It is well-known that both roughness and wettability influence the adhesion of microorganisms to the surface of the material.^62^ It was stated that the rougher the surface, the higher the likelihood that microorganisms adhere to it.^63^ To assess the possibility of such adhesion, Albrektsson and Wennerberg^64^ created a four-level classification of surfaces according to their *S*a parameter. Based on this, all coatings achieved from the systems involving 2 mL/L of EO or constituent and the uncoated substrate^2^ were “minimally rough” (*S*a ∼ 0.5–1.0 μm) or “smooth” in the case of EU (*S*a < 0.5 μm). Regarding the coatings obtained from mixtures involving higher concentrations of EO or constituent, all were classified as “rough” (*S*a > 2 μm). This indicates that for the coatings that are enriched into COB and CA of higher concentrations, the possibility of bacteria adhesion is very high. The rest of the coatings may have a moderate or very low tendency for bacterial adhesion. The effects of wettability and SFE on the adherence of microorganisms to the surface of the biomaterial obtained by different groups are somewhat contradictory.^65,66^ Despite this, Weerkamp et al.^67^ agreed that the hydrophobicity of the surface encourages the adherence of hydrophobic bacteria, and the hydrophilicity of the surface promotes the adherence of hydrophilic strains to the surface of biomaterial. Based on that, it is very unlikely to indicate which of the achieved coatings will be favorable for antibacterial protection of titanium. More systematic and advanced research is needed. Comparing these results with our former works,^2,43,57^ we demonstrated that every additive reduces the wettability of the coatings compared to that of the pure chitosan coating, although there are exceptions. The brightest example was a coating containing CEO, for which the wettability angle for water did not differ significantly from that for chitosan. Moreover, we observed that an increase in the concentration of EO or a constituent in the dispersed system leads to a decrease in the wettability angle values for both water and diiodomethane. We also proved that coatings containing constituents have lower wettability angles than coatings containing EOs, which is a common observation that occurs in each of our works.

Considering all of the data obtained, including the adhesion strength, repeatability, and morphology, we chose all of the developed coatings for further investigation of the resistance to electrochemical corrosion and microbiological properties.

### Electrochemical Corrosion Resistance

Figure 20a shows the OCP values for the investigated samples. The electrochemical behavior of the uncoated titanium was typical for metals, characterized by very well-passivated processes.^68^ The OCP value started at about −0.60 V and gradually increased to 0.02 V after approximately 9 h, and it remained constant throughout the experiment. The OCP values of all coated samples oscillated within a range of about −0.13 to 0.61 V. Stable potential values were achieved between 6 and 10 h for all these coatings. The lowest, but stable, potential value (−0.1 V) was registered for the TH/CS coating achieved from the composition involving 2 mL/L TH (designated as TH2/CS), contrary to the highest potential value, equal to 0.08 V, for the CA/CS coating achieved from the mixture involving 6 mL/L (designated as CA6/CS).

**Figure 20:**
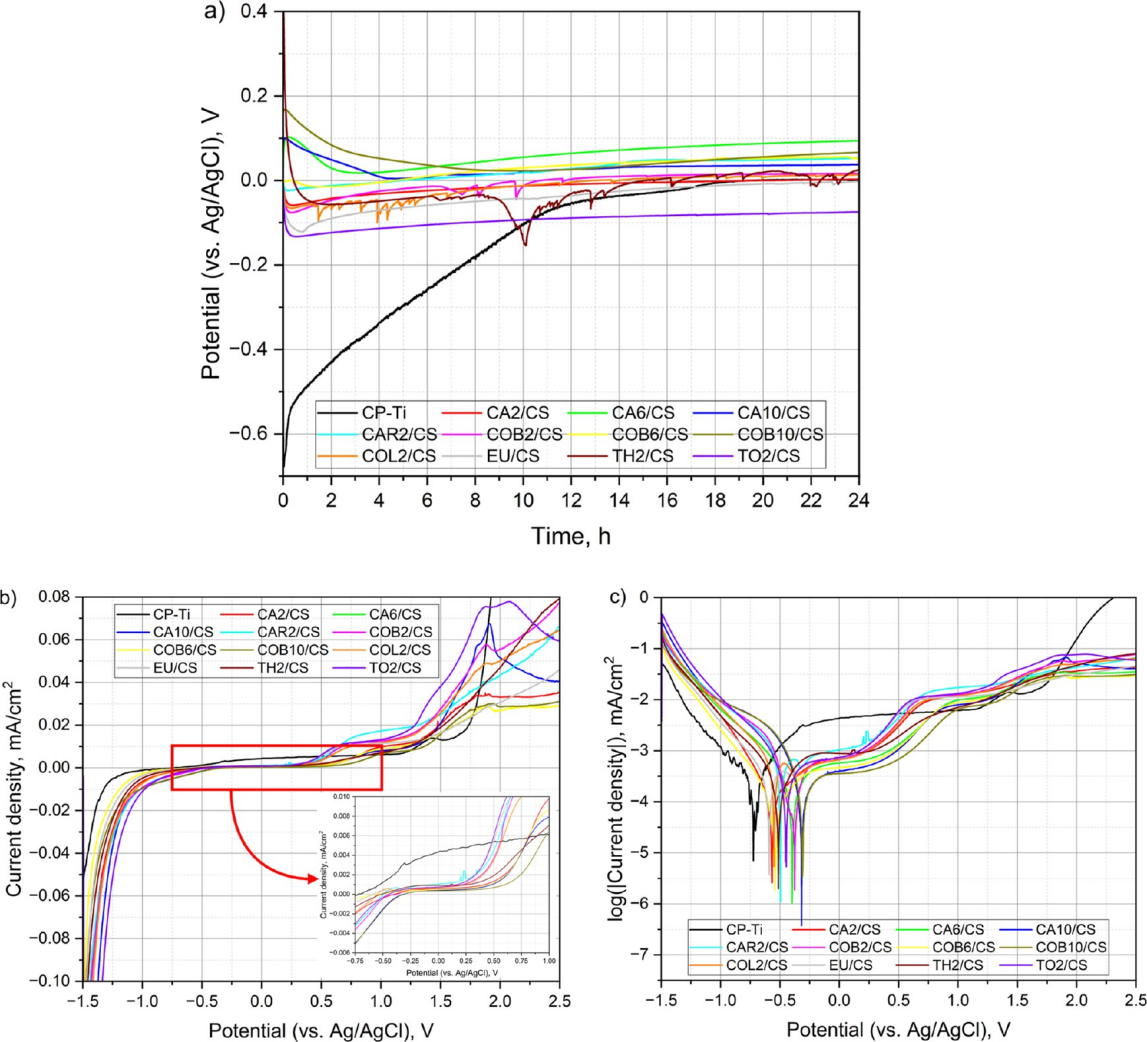
OCP curves, (a) linear sweep voltammetry, and (b) potentiodynamic polarization curves—logarithmic scale (c) of investigation samples. The number after the component in the coating name indicates the content of that component in the EPD dispersed system.

Figure 20b,c presents the polarization curves on the linear scale (Figure 20b) and the logarithmic scale (Figure 20c). It can be observed that the course of all the curves was quite similar on both the anode and the cathode sides, with a large plateau of passivation region (this can be visible more clearly in Figure 20b). The absence of a linear region on the anode and cathode curves in Figure 20c of at least one decade indicated that it is impossible to calculate the corrosion rate from the slope of straight lines. For these kinds of results, the corrosion resistance of the analyzed materials is measured by the current density passing through the passivation area. As the current density values increase, the corrosion rate indicates a lower corrosion resistance.^69^

The bare titanium substrate had the lowest corrosion resistance with the highest current density value equal to 4.57 × 10^–3^ μA/cm^2^. Quite contrary, on the CP-Ti sample, a wide passivation region was observed in the potential range of 0.3–1.2 V. From the potentials above 1.2 V, the anodic current density of the Ti substrate grows at a faster rate, suggesting TiO_2_ passive layer breakdown.^70^ For the majority of the coated samples under investigation, the passivation domain was eminently shorter and comparable, between −0.32 and 0.25 V. The lowest current density equal to 5.60 × 10^–7^ μA/cm2 with the larger passivation regions in the ranges of −0.32–0.56 V and −0.32–0.56 V were recorded for the COB10/CS and CA10/CS coatings, respectively.

The study of electrochemical impedance spectroscopy data (Figures 21a and 22) indicates an improvement in electrochemical corrosion resistance for all coated samples as compared to the bare Ti substrate. Among the investigated, the COB10/CS and CA10/CS coatings showed the highest values of impedance, respectively, 4 and 3× higher than for the CAR2/CS coating, which exhibited the lowest impedance of all of the investigated coatings. A tendency can be observed inside the two groups of coatings, i.e., the CA/CS and COB/CS, namely the correlation between the concentration of CA or COB and the impedance; the impedance arcs become larger with the increasing additive concentration (Figures 21a and 22). The change in the shape of impedance spectra (Figure 21a) and in phase angle–frequency graphs (Figure 22b) imply dissimilarities in the structure of the samples as well as in the electrochemical processes taking place at the phase boundaries and in the bulk of the sample phases. The decrease in the phase angle in the midfrequency range is related to the presence of additional phases in the coating volume and charge transfer processes through these phases. These can be of faradaic (electrochemical reactions) or nonfaradaic (e.g., diffusion, migration, charging of electrical double layers, etc.) type, and both give a contribution to the amplitude of measured current. These properties are also reflected in the arrangement of electrical equivalent circuits (EC), where the more complex the structure of the system and processes occurring, the more complicated the resulting EC becomes. Each component of the EC and each branch can act as an alternative route for the flux of charge carriers. For the uncoated Ti substrate, a simplified Randles circuit [*R*(QR)] was chosen (Figure 21b), with *R*_S_—the electrolyte resistance, CPE_dl_—the nonideal capacitance of the electrical double layer, and *R*_ct_—the resistance for charge transfer. In the case of the samples with the COB2, COB6, and COB10 and COL2 coatings, the equivalent circuit in the form of [*R*(QR)­(QR)] (Figure 21c) was recognized as the most fitting. The CPE_1_ and *R*_1_ represent, respectively, the nonideal capacitance of the coating and the resistance of this layer. Other types of the investigated coatings implicated the application of the Warburg element *Z*_W_ in the EC arrangement, namely the [*R*(QR)­(*Q*[RW])] configuration (Figure 21d). Tables S3 and S4 comprise the values of the fitted parameters of the electrical elements composing the equivalent circuits. Among all the examined samples, the COB10 coating exhibits the highest value of the coating resistance *R*_1_ (i.e., (6.21 ± 0.07)·10^7^ Ω), and this parameter takes higher values as the content of COB increases in the coatings, whereas for the coatings with CA, the resistance *R*_1_ reaches the highest value for the CA6 composition (i.e., (2.13 ± 0.02)·10^7^ Ω). A slightly different pattern can be observed for the CA/CS coatings; namely, the values of admittance recorded for parameter *Z*_W_ significantly decrease with increasing concentration of CA, which suggests that electrochemical reactions occurring at the coating/substrate interface become strongly limited by the diffusion of electroactive species. Among the other coatings, the CA10/CS composition displays the lowest value of the *Z*_W_ parameter, i.e. (7.98 ± 0.31)·10^–6^ S.

**Figure 21:**
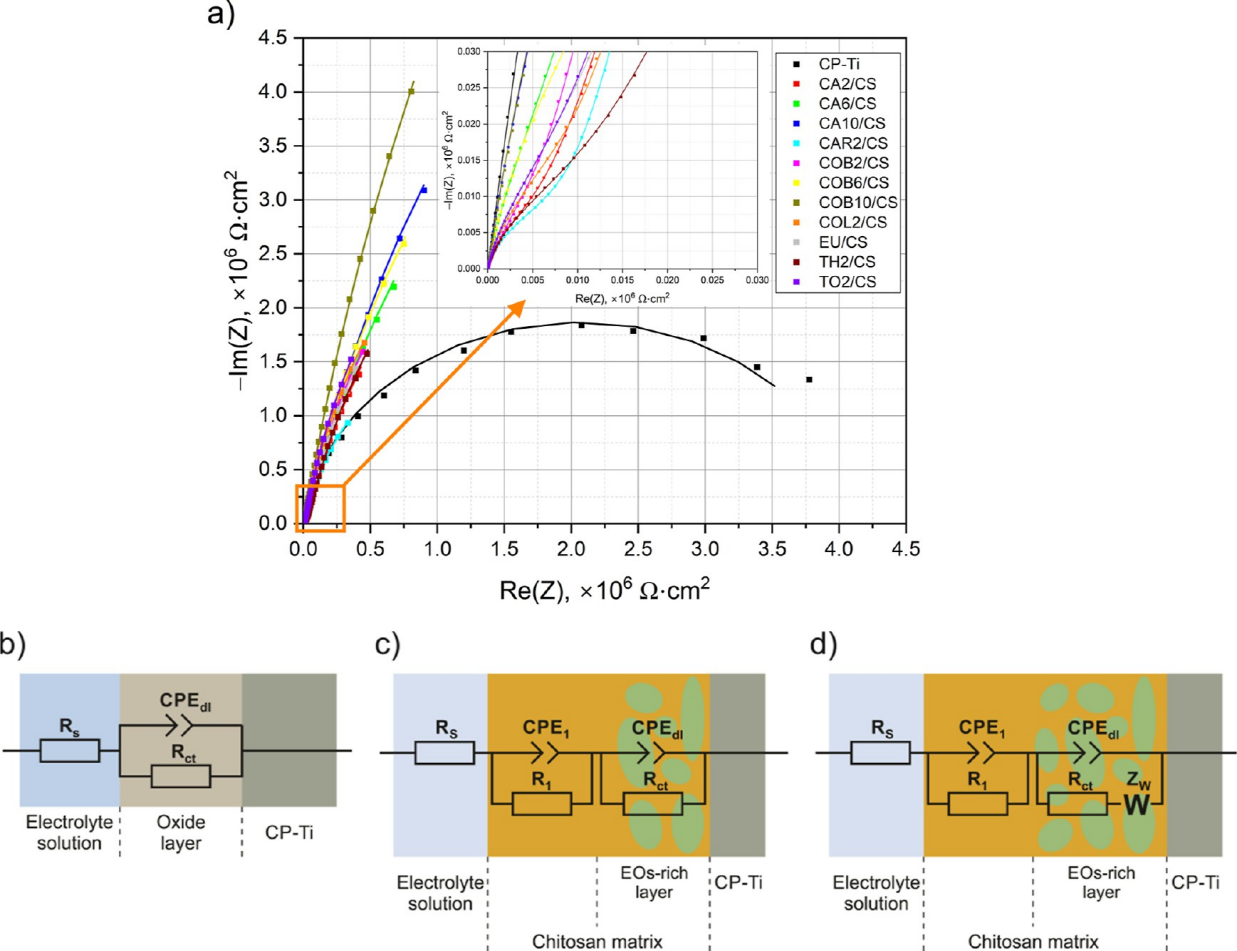
(a) Nyquist plot for EIS data and equivalent circuits for (b) CP-Ti bare substrate, (c) COB2/CS, COB6/CS, COB10/CS, and COL2/CS coatings, and (d) other coatings. Geometric symbols on the Nyquist plot represent experimental data, and the continuous lines are the simulation results based on chosen equivalent circuits.

**Figure 22:**
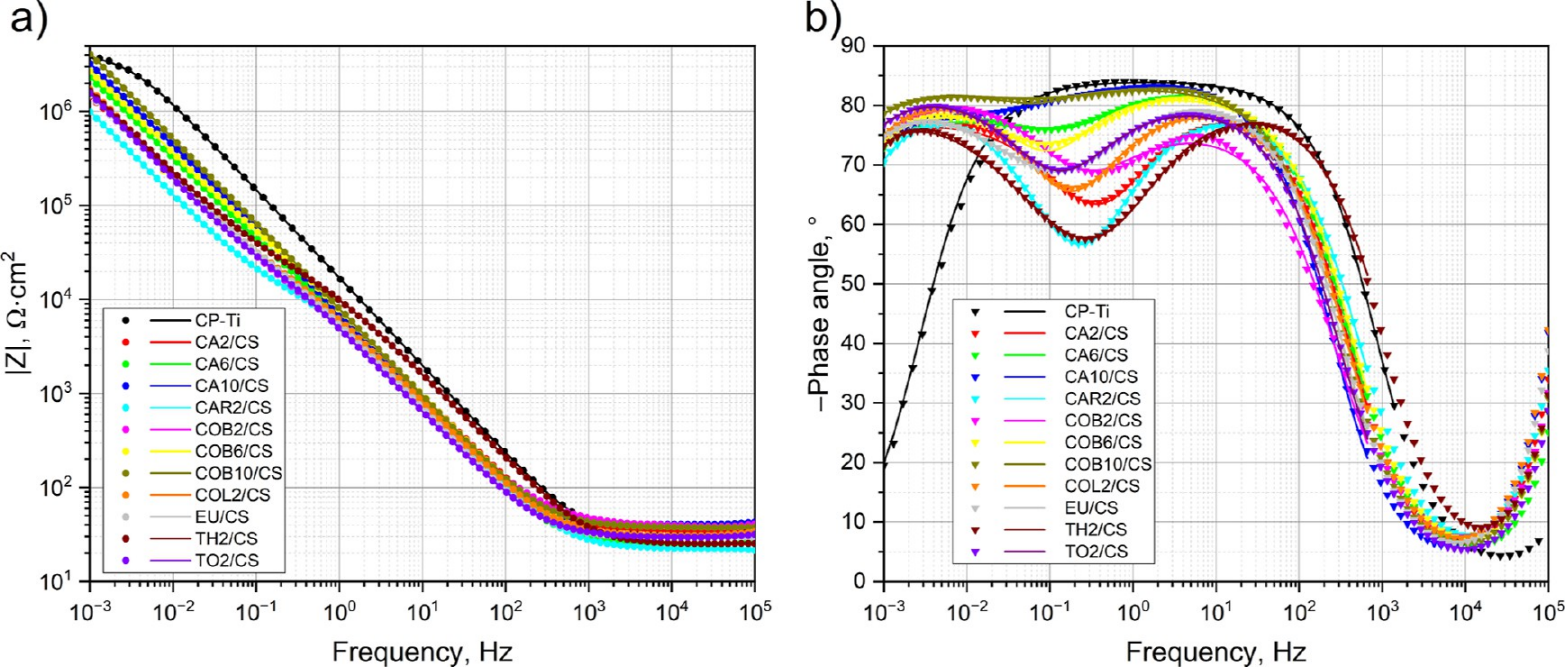
Bode plots for EIS data: (a) modulus of complex impedance and (b) phase angle. Geometric symbols represent experimental data, and the continuous lines are the simulation results based on chosen equivalent circuits.

### Antimicrobial and Antibiofilm Properties

The study evaluated the bactericidal and antibiofilm properties of various surfaces against the viability of two common pathogenic bacterial species: *S. aureus* (Gram-positive bacteria) and *E. coli* (Gram-negative bacteria), see Figure 23.

**Figure 23:**
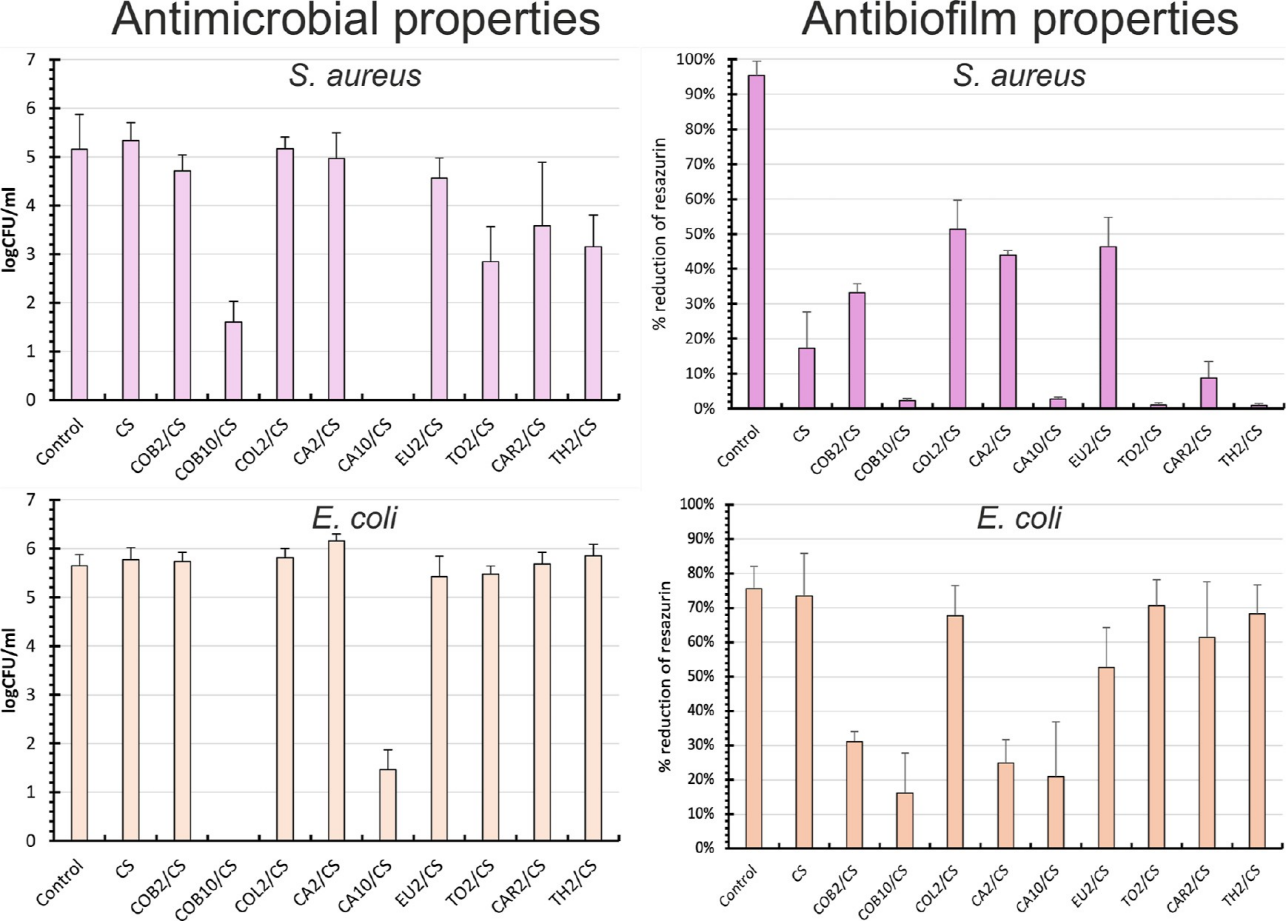
Viability (log CFU/mL) and metabolic activity (%). Biofilms of S. aureus ATCC 25923 and *E. coli* ATCC 25922 developed on CS and CS-based coatings. Viability was determined by the number of colony-forming units (log scale), while metabolic activity was evaluated from the percentage of resazurin reduction.

For *S. aureus* ATCC 25923, the control sample showed an average number of viable bacterial cells at the level of 5.16 ± 0.71 log CFU/mL. The surface of the CS coatings did not exhibit significant bactericidal activity, with a bacterial count at 5.34 ± 0.36 log CFU/mL. The surfaces of the TH2CS and TO2/CS coatings showed antibacterial properties, reducing the number of bacteria to 3.15 ± 0.64 and 2.85 ± 0.72 log CFU/mL, respectively. The surface of the CAR2/CS coating also showed moderate bactericidal activity, with a bacterial number of 3.58 ± 1.31 log CFU/mL. The most effective was the surface of the CA10/CS coating, which significantly eliminated the bacteria cells (0.00 log CFU/mL). The surface of the COB10/CS coating also demonstrated strong bactericidal properties, reducing bacterial cells to 1.60 ± 0.43 log CFU/mL. In contrast, when the additive content in the coating was lower, their antibacterial effectiveness drastically decreased. For the surfaces of the CA2/CS and COB2/CS coatings, the bactericidal effect was almost negligible, with bacterial counts of 4.96 ± 0.50 log CFU/mL and 4.71 ± 0.30 log CFU/mL, respectively. The surfaces of the COL2/CS and EU2/CS coatings exhibited slight antibacterial activity in relation to *S. aureus*, with bacterial counts of 5.17 ± 0.24 and 4.57 ± 0.41 log CFU/mL, respectively.

In terms of the antibiofilm properties, the surface of CS coatings significantly reduced the *S. aureus* biofilm to 17.30% ± 10.36%, indicating strong antibiofilm activity. The surfaces of the TH2/CS and TO2/CS coatings were the most effective, reducing biofilm formation to 0.88% ± 0.58% and 1.07% ± 0.62%, respectively. The CAR2/CS coating also showed strong antibiofilm properties, reducing biofilm to 8.77% ± 4.71%. The CA10/CS coating reduced biofilm to 2.72% ± 0.51%, while the COB10/CS coating also demonstrated strong antibiofilm effects, with a reduction to 2.28% ± 0.60%. However, lowering the antibacterial agent concentration also weakened the antibiofilm properties. For the CA2/CS coating, the biofilm formation remained at 43.95% ± 1.35%, while for the COB2/CS coating, it was 33.21% ± 3.46%. The COL2/CS and EU2/CS coatings showed weaker antibiofilm activity, reducing biofilm to 51.40% ± 8.21% and 46.32% ± 8.49%, respectively.

For the second tested species, *E. coli* ATCC 25922, a middle viable bacterial count of 5.64 ± 0.24 log CFU/mL was observed in the control. Similar to *S. aureus*, the CS coating did not show significant bactericidal activity, with a bacterial count of 5.77 ± 0.25 log CFU/mL. The TH2/CS and CAR2/CS coatings also did not significantly reduce the number of bacteria, with the values of 5.85 ± 0.24 and 5.68 ± 0.24 log CFU/mL, respectively. The TO2/CS coating showed very weak antibacterial activity, reducing the bacterial count to 5.47 ± 0.16 log CFU/mL. The most effective were the CA10/CS coating, which significantly reduced the number of bacteria to 1.47 ± 0.40 log CFU/mL, and the COB10/CS coating, which eliminated the bacteria (0.00 log CFU/mL). Similarly, for this bacterial species, lowering the antibacterial additive concentration markedly reduced the bactericidal properties. The CA2/CS and COB2/CS coatings did not eliminate bacterial cells, with counts of 6.15 ± 0.14 log CFU/mL and 5.74 ± 0.18 log CFU/mL, respectively. The COL2/CS and EU2/CS coatings showed weak or no significant antibacterial effect, with bacterial counts of 5.82 ± 0.18 log CFU/mL and 5.43 ± 0.41 log CFU/mL, respectively.

Regarding antibiofilm activity against *E. coli*, the CS coating did not show a significant effect, with a result of 73.52% ± 12.40%, which is comparable to the control. The TH2/CS and TO2/CS coatings showed weak activity, reducing biofilm formation to 68.28% ± 8.50% and 70.66% ± 7.61%, respectively. The CAR2/CS coating showed moderate antibiofilm properties, reducing the biofilm to 61.42% ± 16.16%. The CA10/CS coating demonstrated strong antibiofilm activity, reducing biofilm formation to 20.95% ± 15.95%, while the CA2/CS coating with a lower essential oil concentration showed a comparable effect, with biofilm formation reduced to 24.93% ± 6.78%. The most effective was the COB10/CS coating, which reduced the biofilm to 16.15% ± 11.64%. The COB2/CS coating with a lower antibacterial agent concentration was much less effective, limiting biofilm growth only to 31.04% ± 3.04%. The COL2/CS and EU2/CS coatings showed weak antibiofilm properties of 67.68% ± 8.76% and 52.68% ± 11.63%, respectively.

### Cytotoxicity Test

The potential cytotoxicity effect of the tested coatings was analyzed by evaluating their influence on the viability of two human cell lines: FaDu (pharyngeal squamous cell carcinoma) and MG-63 (osteoblast-like cells), see Figure 24.

**Figure 24:**
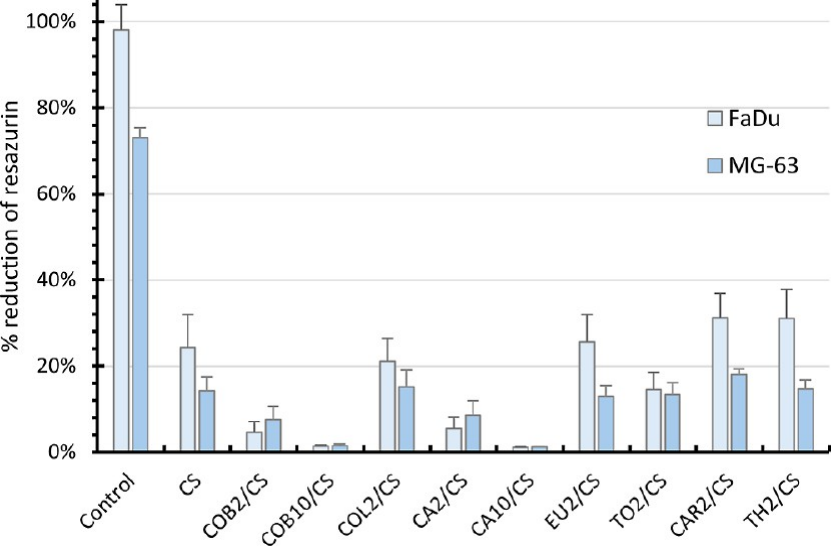
Cytotoxicity of chitosan (CS) and CS-based composite coatings was determined from the metabolic activity of MG-63 and FaDu cell lines using the AlamarBlue assay.

The CS coating essentially lowered the viability of FaDu cells to 24.36% ± 7.60%, indicating strong cytotoxic properties. The CA10/CS and COB10/CS coatings exhibited the highest cytotoxic effects, decreasing cell viability to 1.07% ± 0.13% and 1.40% ± 0.20%, respectively. Slightly weaker but still strong cytotoxic effects were observed for the coatings with lower concentrations of the same antibacterial agent, where the CA2/CS coating reduced cell viability to 5.47% ± 2.58% and the COB2/CS coating to 4.59% ± 2.47%. Strong cytotoxic activity was also observed for the TO2/CS coating, which reduced cell viability to 14.54% ± 4.05%. The COL2/CS and EU2/CS coatings also showed cytotoxic properties, with cell viability levels of 21.16% ± 5.28% and 25.69% ± 6.30%, respectively. In contrast, the CAR2/CS and TH2/CS coatings showed the weakest cytotoxic effects among the tested biomaterials, reducing cell viability to 31.13% ± 6.65% and 31.19% ± 5.69%, respectively.

For the MG-63 cell line, the CS coating reduced cell viability to 14.27% ± 3.28%, indicating strong cytotoxicity. The CA10/CS and COB10/CS coatings showed very strong effects, reducing the viability to 1.18% ± 0.08% and 1.51% ± 0.41%, respectively. In contrast, lowering the antibacterial additive concentration in the CA2/CS and COB2/CS coatings only slightly reduced their cytotoxicity, with viability levels of 7.53% ± 3.13% and 8.65% ± 3.27%, respectively. The COL2/CS and EU2/CS coatings also decreased cell viability to 15.18% ± 3.87% and 12.94 ± 2.51%, respectively. Similar effects were observed for the TH2/CS and TO2/CS coatings, which reduced viability to 14.69 ± 2.03% and 13.41 ± 2.66%, respectively. The CAR2/CS coating also showed strong cytotoxicity, with cell viability at 18.04% ± 1.22%.

To summarize, all tested coatings exhibited strong cytotoxic effects against both cell lines.

## Conclusions

The EPD of the natural composite coatings based on chitosan and containing several essential oils (COB, COL, and TO) and their constituents (CA, EU, TH, and CAR) was developed. This study focused on the determination of the impact of several antibacterial additives on the properties of both dispersed systems and coatings. Based on attained results, the following conclusions were drawn:

- The electrokinetic properties of mixtures utilized for EPD were very similar to each other, showing comparable behavior in a wide range of pH. All dispersed systems were made of droplets of additives in the chitosan matrix despite the initial state of compounds (TH was in the solid form). The droplets had different shapes with respect to the additive used. For COB, CA, and TO, they were circular with smooth edges, and for the rest of the additives, droplets were of irregular shape with frayed edges. In the case of CA, it was observed that it appeared not only as droplets but also as crystalline precipitates.
- Adhesion strength of the coatings was significantly influenced by the content of antibacterial agent in the mixture used for EPD. For TO and COL, it was observed that a concentration greater than 2 mL/L resulted in very poor adhesion strength. On the other hand, in the case of COB and in the excess of CA, the adhesion strength is nearly the same in the range of 2–10 mL/L of additive content in the dispersed mixture.
- The concentration of the additive in the dispersed system also had an impact on the microstructure and morphology of the coatings. As the amount of EO or constituent in the system increases, the number of droplets and their size also increase. The appearance of the droplets in the chitosan matrix also depended on the type of additive. The constituents were harder to observe than EOs because of their solubility in ethyl alcohol or water. FT-IR investigation of the obtained coatings proved the occurrence of all additives in the coatings. Moreover, it showed the formation of hydrogen bonds between the main constituents of EOs and chitosan, which altered the structure of the biopolymer matrix.
- The type of additive and its concentration have an impact on the roughness parameters, surface development, and wettability of the obtained coatings. Mostly the additives increased the roughness of the coatings (the only exception being EU) in comparison to the pure chitosan coating. In the case of CA and COB, it was observed that increasing the concentration led to an increase in both the roughness of the coating and the surface development. All additives decreased wettability in comparison to that of pure chitosan coating. For the CA/CS and COB/Cs coatings, it was observed that the lower the concentration, the higher the WA. All additives increased the SFE compared to the chitosan coating. SFE increased with the increasing concentration of antibacterial additive in the EPD dispersed systems. For all coatings the polar part of SFE was smaller than the dispersive part but much larger than for the chitosan coating.
- The corrosion resistance of all coated titanium substrates studied was superior to that of bare titanium. Corrosion resistance was observed to be highest for two coatings: CA10/CS and COB10/CS, which were directly related to the concentration of CA for the CA10/CS coating and COB for the COB10/CS coating.
- All coatings exhibited relatively good antibiofilm properties against both *S. aureus* and *E. coli*, with better behavior against the first one. It was observed that the coatings achieved from the mixtures involving 10 mL/L of CA or COB acted exceptionally well against both bacteria strains regardless of the time of incubation. All the analyzed coatings were highly cytotoxic against both the FaDu and MG-63 cell lines.

The present work provides insight into the further development of chitosan coatings with natural antimicrobial agents. The most promising additives used in this study are COB and CA. Coatings containing those substances exhibited superior adhesion strength to the substrate regarding concentration in the emulsion used for EPD and had a high microbicidal potential with respect to examined bacteria strains. The occurrence of high cytotoxicity of the developed coatings clearly shows that more systematic research is needed on the distribution and volume fraction of antimicrobial agents in the chitosan matrix.

## Supplementary Material



## Data Availability

The raw and processed data required to reproduce results presented in this publication are available on https://doi.org/10.58032/AGH/UQLZGE.

## Notes

Detailed FT-IR spectra of CS-based coatings (Tables S1 and S2) and fitted parameters of elements of EC (Tables S3 and S4) (PDF)
