# Supplementary material for: Impact of Various Essential Oils or Their Pure Components on the Selected Properties and Microbicidal Potential of Chitosan-Based Coatings
Source: Langmuir. 2026 Mar 16;42(12):8817–37. doi: 10.1021/acs.langmuir.6c00099 (PMC13362200; doi:10.1021/acs.langmuir.6c00099)
Supplement: Supplementary file 1 [file la6c00099_si_001.pdf]

Supplementary materials

Impact of various essential oils or their pure components on the selected properties and microbicidal potential of chitosan-based coatings

Mikołaj Mielczarek[1], Jakub Marchewka[2], Alicja Łukaszczyk[3], Katarzyna Biegun-Drożdż[4], Kamil Drożdż[4], Tomasz Gosiewski[4], Maciej Sitarz[2], Monika Brzychczy-Włoch[4], Tomasz Moskalewicz[1]

[1] AGH University of Krakow, Faculty of Metals Engineering and Industrial Computer Science, Czarnowiejska 66, 30-054 Kraków, Poland

[2] AGH University of Krakow, Faculty of Materials Science and Ceramics, Mickiewicza Av. 30, 30-059 Kraków, Poland

[3] AGH University of Krakow, Faculty of Foundry Engineering, Mickiewicza Av. 30, 30-059 Kraków, Poland

[4] Jagiellonian University Medical College, Faculty of Medicine, Chair of Microbiology, Department of Molecular Medical Microbiology, Czysta 18, 31-121 Kraków, Poland

Corresponding authors: Prof. Tomasz Moskalewicz, tel.: +48 126174527, e-mail: tmoskale@agh.edu.pl, MSc. Eng. Mikołaj Mielczarek, e-mail: mmielcz@agh.edu.pl,

List of content: Detailed FT-IR spectra of CS-based coatings (Tables S1 and S2); Fitted parameters of elements of equivalent circuits (Tables S3 and S4)

Table S1. Detailed FT-IR spectrum analysis for the chitosan coating as well as the chitosan coatings with cinnamon essential oils (bark and leaf type) and their components

**band position** [cm^{-1}] **and characteristics**					**type of vibration**
**pure CS coating**	**COB/CS coatings**	**CO>/CS coatings**	**CA/CS coatings**	**EU/CS coatings**	
3435 s, br	3441 s, br	3423 s, br	3438 s, br	3435 s, br	ν O-H
3373 s, br	3366 s, br	3366 s, br	3367 s, br	3366 s, br	ν N-H in $-NH_2$
3300 s, br	3298 s, br	3301 s, br, sh	nv	3302 s, br, sh	ν N-H in CH_3CONH-
2957 m	nv	nv	nv	nv	ν C-H
2920 m	2918 m	2923 m	2918 m	2918 m	ν C-H
2873 m	2876 m	nv	2881 m	2874 m	ν C-H
1657 m	nv	1642 m	nv	1638 m	ν C=O in CH_3CONH-
1569 m	1560 m	1564 m	1561 m	1563 m, sh	δ N-H
1420 m	1412 m	1412 m	1411 m	1418 m	δ C-H in (ring)-CH_2OH
1378 m	1378 m	1383 m	1371 m	1381 m	δ C-H in ring
1323 m	1318 m	1326 m	1315 m	1323 m	ν C-N
1257 w	1259 w	1261 w	1259 w	1263 w	ν C-C in ring
1152 s	1153 s	1152 m	1151 s	1152 m	ν C-O in (ring)-OH
1085 vs	nv	1068 vs	nv	1067 vs	ν C-O-C in ring
1032 s	1034 s	1031 s	1031 s	1033 s	ν C-O-C between rings
948 m	nv	948 m, sh	nv	945 m, sh	ν C-C
891 w	898 m	898 m	898 m	898 m	δ C-H
824 w	nv	nv	nv	nv	δ C-H
815 w	nv	nv	nv	nv	δ C-H
798 w	nv	nv	nv	nv	δ C-H
781 w	nv	nv	nv	nv	δ C-H
662 m	nv	661 w	nv	655 w	δ N-H in $-NH_2$
614 m	nv	nv	nv	nv	δ N-H in CH_3CONH-
575 m	nv	nv	nv	nv	δ C=O in CH_3CONH-

Abbreviations: bands intensity: vs - very strong, s - strong, m - medium, w - weak; bands shape: br - broad, sh - shoulder; type of vibration: ν - stretching, δ - bending, s - symmetric, as - asymmetric; nv - band not visible.

Table S2. Detailed FT-IR spectrum analysis for the chitosan coating as well as the chitosan coatings with thyme essential oil and its components.

**band position** [cm^{-1}] **and characteristics**				**type of vibration**
**pure CS coating**	**TO/CS coatings**	**CAR/CS coatings**	**TH/CS coatings**	
3435 s, br	3449 S, BR	3456 S, BR	3438 S, BR	ν O-H
nv	3388 S, BR	3387 S, BR, SH	3385 S, BR	ν O-H
3373 s, br	3366 S, BR	3368 S, BR	3364 S, BR	ν N-H in $-NH_2$
3300 s, br	3300 S, BR	3302 S, BR	3302 S, BR	ν N-H in CH_3CONH-
2957 m	2963 M, SH	nv	2961 M, SH	ν C-H
2920 m	2928 M	2925 M	2927 M	ν C-H
2873 m	nv	nv	nv	ν C-H
1657 m	1652 M<br>1646 M<br>1637 M, SH	1640 M	1652 M, SH<br>1645 M<br>1636 M	ν C=O in CH_3CONH-
1569 m	1567 S	1564 S	1559 S	δ N-H
1420 m	1418 M	1413 M	1411 S	δ C-H in (ring)-CH_2OH
1378 m	1384 M	1385 M	1381 S	δ C-H in ring
1323 m	1325 M	1324 M	1325 M	ν C-N
1257 w	1252 W	1260 W	1261 W	ν C-C in ring
1152 s	1150 S	1151 S	1150 M	ν C-O in (ring)-OH
1085 vs	1079 VS	1071 VS	1066 VS	ν C-O-C in ring
1032 s	1038 VS	1033 VS	1028 VS	ν C-O-C between rings
948 m	948 M	947 W, SH	944 W, SH	ν C-C
891 w	896 M	898 W	896 W	δ C-H
824 W	nv	nv	nv	δ C-H
815 W	nv	nv	nv	δ C-H
798 W	nv	nv	nv	δ C-H
781 W	nv	nv	nv	δ C-H
662 M	656 W	654 W	654 W	δ N-H in $-NH_2$
614 m	nv	nv	nv	δ N-H in CH_3CONH-
575 m	nv	nv	nv	δ C=O in CH_3CONH-

abbreviations: bands intensity: vs - very strong, s - strong, m - medium, w - weak; bands shape: br - broad, sh - shoulder; type of vibration: ν - stretching, δ - bending, s - symmetric, as - asymmetric; nv - band not visible.

Table S3. Fitted parameters of elements of the equivalent circuits

Element of EC		Sample					
		**CP-Ti**	**CA2/CS**	**CA6/CS**	**CA10/CS**	**CAR2/CS**	**COB2/CS**
R_S	Ω	32.51 ± 0.34	37.77 ± 0.26	25.52 ± 0.14	41.66 ± 0.15	22.69 ± 0.05	43.15 ± 0.53
CPE_1	Y, $\times 10^{-5}$ S	-	5.80 ± 0.12	3.90 ± 0.02	7.51 ± 0.34	10.42 ± 0.08	5.84 ± 0.07
	n	-	0.88 ± 0.01	0.89 ± 0.01	0.99 ± 0.05	0.92 ± 0.01	0.91 ± 0.01
R_1	$\times 10^7$ Ω	-	1.65 ± 0.02	2.13 ± 0.02	1.41 ± 0.04	0.51 ± 0.09	1.33 ± 0.02
CPE_{dl}	Y, $\times 10^{-5}$ S	1.05 ± 0.01	4.41 ± 0.12	10.09 ± 0.03	3.73 ± 0.81	4.45 ± 0.03	9.53 ± 0.28
	n	0.94 ± 0.01	0.92 ± 0.01	0.99 ± 0.01	0.91 ± 0.01	0.89 ± 0.01	0.81 ± 0.01
R_{ct}	$\times 10^3$Ω	4131.72 ± 36.61	5.98 ± 0.21	3.49 ± 0.54	10.07 ± 1.09	9.11 ± 0.44	6.71 ± 0.48
Z_W	$\times 10^{-6}$ S	-	384.27 ± 68.94	191.92 ±11.94	7.98 ±0.31	476.74 ± 26.22	-
χ^2	$\times 10^{-3}$	14.49	9.12	3.46	3.15	7.18	29.69

Table S4. Fitted parameters of elements of the equivalent circuits

Element of EC		Sample					
		**COB6/CS**	**COB10/CS**	**COL2/CS**	**EU2/CS**	**TH2/CS**	**TO2/CS**
R_S	Ω	30.47 ± 0.28	40.04 ± 0.28	31.63 ± 0.14	29.71 ± 0.07	23.46 ± 0.28	30.51 ± 0.08
CPE_1	Y, ×10^{-5} S	3.33 ± 0.04	2.48 ± 0.02	5.81 ± 0.03	7.55 ± 0.14	7.72 ± 0.23	7.16 ± 0.12
	n	0.90 ± 0.01	0.92 ± 0.01	0.91 ± 0.01	0.93 ±0.01	0.94 ±0.01	0.93 ±0.01
R_1	×10^7 Ω	2.44 ± 0.03	6.21 ± 0.07	1.47 ± 0.07	1.31 ± 0.06	1.09 ± 0.09	1.42 ± 0.07
CPE_{dl}	Y, ×10^{-5} S	8.41 ± 0.27	31.21 ± 3.08	6.12 ± 0.05	5.81 ± 0.11	1.84 ± 0.03	7.75 ± 0.19
	n	0.96 ± 0.01	0.99 ± 0.05	0.88 ± 0.01	0.90 ±0.01	0.89 ±0.01	0.87 ±0.01
R_{ct}	×10^3Ω	14.24 ± 1.27	6.34 ±1.54	11.24 ± 0.21	12.36 ±0.28	13.02 ± 0.23	12.30 ± 0.29
Z_W	×10^{-6} S	-	-	-	122.61 ± 7.71	65.83 ± 3.29	465.71 ± 89.73
χ^2	×10^{-3}	13.67	10.86	3.67	1.16	6.23	1.78
